# Oleogels for the ocular delivery of epalrestat: formulation, in vitro, in ovo, ex vivo and in vivo evaluation

**DOI:** 10.1007/s13346-024-01560-7

**Published:** 2024-05-23

**Authors:** Axel Kattar, Maria Vivero-Lopez, Angel Concheiro, Rajeev Mudakavi, Anuj Chauhan, Carmen Alvarez-Lorenzo

**Affiliations:** 1 Departamento de Farmacología, Farmacia y Tecnología Farmacéutica, Facultad de Farmacia, Instituto de Materiales (iMATUS) and Health Research Institute of Santiago de Compostela (IDIS), Universidade de Santiago de Compostela, Santiago de Compostela, 15782 Spain; 2 Department of Chemical Engineering, Colorado School of Mines, Golden, CO 80401 USA

**Keywords:** Oleogel, Epalrestat, Ocular delivery, Diabetes, Topical administration, Ocular biodistribution

## Abstract

The ocular administration of lipophilic and labile drugs such as epalrestat, an aldose reductase inhibitor with potential for diabetic retinopathy treatment, demands the development of topical delivery systems capable of providing sufficient ocular bioavailability. The aim of this work was to develop non-aqueous oleogels based on soybean oil and gelators from natural and sustainable sources (ethyl cellulose, beeswax and cocoa butter) and to assess their reproducibility, safety and efficiency in epalrestat release and permeation both ex vivo and in vivo. Binary combinations of gelators at 10% w/w resulted in solid oleogels (oleorods), while single gelator oleogels at 5% w/w remained liquid at room temperature, with most of the oleogels displaying shear thinning behavior. The oleorods released up to 4 µg epalrestat per mg of oleorod in a sustained or burst pattern depending on the gelator (approx. 10% dose in 24 h). The HET-CAM assay indicated that oleogel formulations did not induce ocular irritation and were safe for topical ocular administration. Corneal and scleral ex vivo assays evidenced the permeation of epalrestat from the oleorods up to 4 and 2.5 µg/cm^2^ after six hours, respectively. Finally, the capacity of the developed oleogels to sustain release and provide significant amounts of epalrestat to the ocular tissues was demonstrated in vivo against aqueous-based niosomes and micelles formulations loaded with the same drug concentration. Overall, the gathered information provides valuable insights into the development of oleogels for ocular drug delivery, emphasizing their safety and controlled release capabilities, which have implications for the treatment of diabetic neuropathy and other ocular conditions.

**Graphical abstract:**

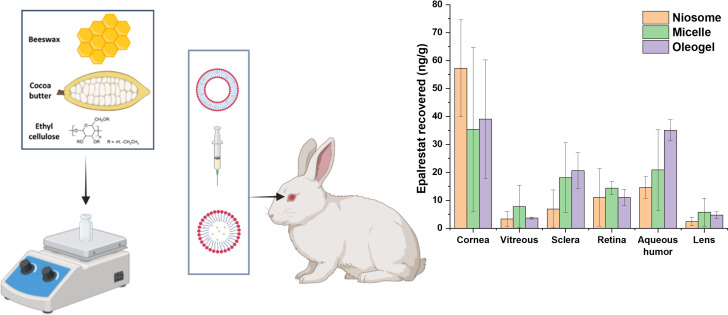

**Supplementary Information:**

The online version contains supplementary material available at 10.1007/s13346-024-01560-7.

## Introduction

A huge increase in eye care and treatment is expected to take place over the next years due to the prevalence of diabetes in an ageing population [1, 2]. Blood glucose levels in diabetic patients can be high enough to activate an uncommon glucose conversion pathway: the polyol pathway driven by the enzyme aldose reductase triggering the conversion of glucose to sorbitol [3]. Sorbitol accumulation in the cells leads to oxidative and osmotic stress [4, 5], cell death [6], cataracts, and ultimately vision loss.

Patients suffering from diabetic ocular diseases demand efficient ophthalmic drug delivery systems capable of overcoming the numerous barriers of the eye [7]. Topical administration is the most convenient delivery method for the treatment of chronic ocular diseases as it does not require the assistance of any medical personnel, putting the patient in charge of their own treatment. Eye drops are the most common form of topical administration, but they have a severe drawback when it comes to bioavailability due to tear clearance [8]. To overcome tear clearance and promote the access of drugs to the therapeutic site of the eye different alternatives, such as medicated contact lenses [9], nanocarriers like liposomes [10], niosomes [11], cyclodextrin aggregates [12], microemulsions [13], intraocular injections [14] or implanted hydrogels [15] are being investigated. Although topical formulations have brought their own challenges [16], preclinical studies have already evidenced the potential of topical administration to reach anterior and posterior eye segment, specially when non-aqueous vehicles are used [17].

Epalrestat has demonstrated being useful for the treatment of diabetes-related diseases since acts as an aldose reductase inhibitor, stopping the conversion of glucose to sorbitol in high glucose environments [18, 19]. Its safety has been studied in vitro on retinal pigment epithelial cells [20] and in vivo by oral administration on albino rabbits [21]. Further in vivo studies have looked at the pharmacokinetics of epalrestat in rabbits [22] and the effect of epalrestat on pulmonary fibrosis in rats [23]. However, its hydrophobic nature makes difficult its ocular administration. So far, only contact lenses [9] and niosomes [11] have been designed as platforms for sustained delivery on the ocular surface or to promote cornea and sclera penetration, respectively. While these systems successfully encapsulated epalrestat they were not yet investigated in vivo.

Oleogels are made out of a gelator and an oil that, after being melted together, form semisolid formulations of tunable viscosity and spreadability on the corneal epithelium [17, 24]. Oleogels can be designed to remain solid or become fluid when they come into contact with warm body temperatures, making them ideal for ocular drug delivery either as eye drops or as rod inserts [25]. Gels prepared in non-aqueous vehicles are more biocompatible than hydrogels as they do not need pH and osmolarity adjustments neither preservatives and surfactants, reducing the risk of ocular irritation and inflammation [17, 26, 27]. Another advantage of oleogels is that they have a prolonged residence time on the eye surface compared to hydrogels and aqueous suspensions due to their ability to form a protective film on the ocular surface, reducing the rate of drug clearance. This is particularly important for patients with limited dexterity or who have difficulty instilling eye drops, as they can space successive installations. Furthermore, oleogels are able to retain a wide range of drugs, including hydrophobic and hydrophilic compounds [25, 28]. Research on oleogels for ocular drug delivery is gathering momentum, although biocompatibility of all components and avoidance of blurry vision should be considered [26, 28–31]. Oleogels based on soybean oil have also been recently shown suitable for punctal occlusion and thus to prolong drug (cyclosporine A) permanence on the ocular surface and to increase drug accumulation in cornea, conjunctiva and sclera [32].

We hypothesize that oleogels capable of releasing epalrestat in a sustained manner to the eye, by forming a coat of loaded gel on the ocular surface, may be a valuable drug delivery platform in the context of diabetic ocular diseases. So, the aim of this work was to develop different oleogels based on soybean oil using gelators from natural and sustainable sources and to assess their reproducibility, safety and efficiency in epalrestat release and permeation both ex vivo and in vivo. Ethyl cellulose, beeswax and cocoa butter were chosen as the gelators and tested in separate and in binary combinations. Main criteria to develop the formulations were adequate viscosity to be topically administered and concentration of epalrestat sufficiently high to reach therapeutic concentrations in the posterior segment of the eye while minimizing local side effects. It has been previously shown in HET-CAM tests that free epalrestat is highly irritant for conjunctiva-like vasculature [6]. Thus, 0.2 mg/mL of epalrestat was chosen as a compromise concentration between efficacy and safety. The most adequate oleogel formulation was then compared in vivo to niosomes [11] and micelles loaded with the same concentration of epalrestat using New Zealand white rabbits. Micelles were chosen as “formulation of reference” because of their proven ability to increase ocular surface residence time and corneal penetration of hydrophobic drugs in rabbit model experiments, [33–35]. The micelles chosen to fill this comparative role were made of Pluronic^®^ F127, a polymer of poly (ethylene oxide)-poly(propylene oxide)-poly(ethylene oxide) (PEO-PPO-PEO) regarded as safe, which have been shown in in vivo ocular drug delivery experiments on rabbit models to notably enhance drug ocular bioavailability [33, 36, 37]. Micelles may host epalrestat in the hydrophobic core, since direct solution of epalrestat in water is not feasible.

In vivo experiments are an essential step in the bench to bedside process of drug formulation development. While the experiments on ex vivo tissues and the toxicity tests are very valuable to reduce the number of potential formulations fit for human testing, ex vivo and in ovo experiments are not yet able to encompass all the variable influencing the absorption and transport of drugs in the eye, thus rendering animal experiments unavoidable. Thus, since there is still a lack of in vitro models that can predict in vivo performance [38], the objective of the preclinical test in rabbits was three-fold: (i) to determine the safety and tolerance of the non-aqueous (oleogel) and aqueous (niosomes and micelles) formulations, (ii) to monitor the epalrestat levels in the tear fluid, and (iii) to measure the accumulation of epalrestat in the different tissues of the eye. The rabbit model is considered to be more appropriate than the rodent model for clinical translation as relevant pharmacokinetic parameters such as clearance rate and distribution are similar to the human eye [39–43]. The evidence provided by experiments on rabbit models balance the reliability of the data gathered and translation to humans while complying with ethical guidelines and cost requirements.

## Materials and methods

### Materials

Soybean oil (ThermoScientific, Kandel, Germany), ethyl cellulose (63 mPa.s, 5% in Toluene/EtOH 80:20, 25 °C, 10 s^− 1^; 48.7% w/w ethoxyl content) (Sigma-Aldrich, Louisville, KY, USA), beeswax (Vabneer, Heibei, China), cocoa butter (BambooStory, Peru), polysorbate 60 (Tween 60, HLB 14.9, 1311.7 g/mol, Sigma Aldrich, Buchs, Switzerland), 1,2-di-O-octadecenyl-3-trimethylammonium propane (chloride salt) (DOTMA, 670.58 g/mol, Avanti, Alabaster, AL, USA), epalrestat ({(5Z)-5-[(2E)-2-methyl-3-phenylprop-2-en-1-ylidene]-4-oxo-2-sulfanylidene-1,3-thiazolidin-3-yl}acetic acid; 319.4 g/mol, TCI, Tokyo, Japan; predicted solubility in water 6.37 mg/L, pKa 3.7, and LogP 3.09 from https://go.drugbank.com/drugs/DB15293), polyoxyethylene (20) sorbitan monooleate (Tween 80, HLB 15, 1310 g/mol, Sigma Aldrich, Switzerland), disodium hydrogen phosphate dihydrate (VWR Chemicals, Briare, France), phosphate buffered saline (PBS; NaCl 137 mM, KCl 2.7 mM, Na_2_HPO_4_ 10 mM and KH_2_PO_4_ 1.8 mM) (Life Tecnologies Co., Carlsbad, CA, USA), ethanol (VWR Chemicals, Briare, France), methanol (Fisher Scientific Loughborough, UK), Kolliphor^®^ P 407 (Pluronic^®^ F127, BASF ChemTrade GmbH, Burgbernheim, Germany), Propofol Lipuro^®^ 10 mg/mL (B. Braun vetcare, Tuttlingen, Germany), pentobarbital sodium Euthasol (Dechra, Barcelona, Spain). Simulated lacrimal fluid (SLF) was made with 0.68 g sodium chloride (Labkem, Barcelona, Spain), 0.22 g sodium bicarbonate (Merck, St Louis, MO, USA), 0.008 g calcium chloride dihydrate (Merck, Darmstadt, Germany) and 0.14 g potassium chloride (Panreac, Castellar del Vallès, Spain) [44] in 100 mL distilled water and the pH was adjusted to 7.5.

### Oleogels formulation

Soybean oil was chosen as the non-aqueous vehicle due to its biocompatibility and high smoke point, allowing for the heating to high temperatures to melt the gelling agents. Three different gelators were chosen: ethyl cellulose as a polymeric gelator, beeswax as a non-lipid based gelator, and cocoa butter as a lipid-based gelator [24]. Oleogels preparation temperature was based on the differential scanning calorimetry (DSC) scans recorded for all excipients and epalrestat in a DSC Q100 TA Instruments (New Castle, DE, USA) by heating samples (1–3 mg) in non-sealed aluminum pans from 40ºC to 200ºC, cooling to 0 ºC, and heating again up to 200ºC, at 10 ºC/min. Stability of epalrestat during heating was evaluated by processing again the same epalrestat-containing pan (after the cyclic heating up to 200ºC) from 40ºC to 350ºC, cooling to 0 ºC, and finally heating again up to 350ºC, at 10 ºC/min. DSC scans of fresh (non-preprocessed) epalrestat were also recorded from 40ºC to 350ºC, cooling to 0 ºC, and heating again up to 350ºC, at 10 ºC/min.

The oleogels were prepared by melting the gelators according to the compositions in Table 1 and adding epalrestat in 1 mL soybean oil at 60 °C (B, C, BC) or 170 °C (E, EB, EC) under 100 rpm magnetic stirring. The single gelator oleogels (B, C and E) were only loaded with 5% w/w epalrestat, while the binary gelator oleogels were loaded with 5, 10 or 30% w/w epalrestat. The temperature of 170 °C was used only when ethyl cellulose was used. Once the components melted and the resulting mixture was homogenous, it was loaded into a 3 mL syringe and cooled to room temperature. Then, 0.1 mL of each formulation was extruded through a 22-gauge needle onto the surface of water, SLF or 1% Tween 80 aqueous solution to form an oleorod. The oleogels can be stored in the syringe at room temperature away from light. Morphological analysis and drug incorporation and dispersion in the oleogels was done by imaging under 4x magnification. Images were taken on an Olympus CKX53 microscope equipped with an Olympus EP50 digital camera (Shinjuku, Tokyo, Japan). Preparation and all subsequent experiments carried out with epalrestat were taken protected from light exposition to prevent drug degradation.

**Table 1:** Oleogels (viscous liquid) and oleorods (solid-like) formulation compositions

Code	Ethyl cellulose % (w/w)	Beeswax % (w/w)	Cocoa butter % (w/w)	Appearance
B	0	5	0	Oleogel
C	0	0	5	Oleogel
E	5	0	0	Oleogel
EB	5	5	0	Oleorod
EC	5	0	5	Oleorod
BC	0	5	5	Oleorod

### Micelles formulation

The micelle formulation was prepared as reported before [33] with minor modifications to encapsulate 0.2 mg/mL epalrestat. Briefly, 10 mM Pluronic^®^ F127 micelles were prepared by dispersing 12.6 w/w Pluronic^®^ F127 in sterile PBS at 300 rpm for 5 h in an ice bath to prevent gelling. Once the copolymer was completely dispersed, epalrestat was added, and the mixture was left to stir for another 5 h at 300 rpm in an ice bath. The final dispersion, named F127, was kept at 4 °C until use.

### Niosomes formulation

Niosomes were prepared according to a method previously reported [11]. Briefly, polysorbate 60 (67 mg), DOTMA (2.57 mg) and cholesterol (8.3 mg) were dissolved in 2 mL of ethanol. Epalrestat (2 mg) was dissolved in 500 µL of ethanol and introduced into the flask. The organic solvent was removed using a rotary evaporator at 70 °C under 50 mbar pressure, resulting in a thin film. This film was subsequently desiccated for 30 min. Then, 10 mL of sterile PBS was added to this flask and the film dislodged from the walls through ultrasonication for 30 min. To create niosomes, the solution was sonicated for 90 s at 20% amplitude using a Branson Digital Sonifier 450 (Marshall Scientific, Hampton, NH, USA). This process yielded epalrestat-loaded niosomes in PBS named TCD5.

### Micelles and niosomes characterization

Formulations were characterized in terms of size, polydispersity index (PDI), and surface charge. The particle size and polydispersity index of 10 mM Pluronic^®^ F127 micelles were measured in ultrapure water at 20 °C and with 10 s equilibration time using a Zetasizer Pro-Blue (Malvern Instruments, UK; detector angle 173º, back scatter). Folded capillary cuvettes (DTS1070) were used for the measurements and the values taken were recorded by number.

### Epalrestat loading efficiency

The loading efficiency (LE%) was measured differently for each system. The loading efficiency of the niosomes was measured by dialyzing the niosomes for 30 min in ultrapure water with 1 vol% Tween 80 and analyzing the medium with HPLC [11]. The dialysis membrane had a molecular weight cutoff of 12,000 Da and an effective dialysis area of 4.2 cm^2^. The concentration was confirmed by lysing the niosomes with methanol and measuring the concentration of the drug encapsulated by HPLC. The efficiency was then calculated with Eq. 1:

1 $$ LE\%=1- \frac{amount\, of drug out\, of\, the\, dyalisis\, membrane}{total\, amount\, of\, drug}*100$$

The loading efficiency of the micelles was measured by ultracentrifugation of the micellar suspension at 4 °C and 14,000 rpm for 30 min and analyzing the supernatant by HPLC. The efficiency was then calculated with Eq. 2:

2 $$ LE\%=1- \frac{amount\, of\, drug in\, the\, supernatant}{total\, amount\, of\, drug}*100$$

The loading efficiency of the oleogels was qualitatively described by the presence or absence of epalrestat crystals in the oleogel at 4x magnification. If there was an absence of epalrestat crystals in the oleogel the loading efficiency was determined to be 100%.

### Viscosity

Rotational rheology of oleorods and oleogels as well as niosomes TDC5 and micelles F127 was performed on an AR-1000 N Rheometer (TA Instruments, Surrey, UK). The geometry used was a 4 cm cone solvent trap with 1.58 degree angle, and the gap was 50 μm. The experiments were conducted at 20 and 35 °C from 0.05 to 1000 s^− 1^, recording 100 sampling points.

### Epalrestat release

EB, EC and BC oleorods (0.1 mL) were deposited on top of 10 mL release medium inside a closed off vial. Three media were tested: distilled (DI) water, 1% v/v Tween 80 in water, and SLF. Release was tested at 20 °C and at 37 °C. 1 mL of release medium was drawn at predefined timepoints, to be analyzed at 400 nm by UV-VIS spectroscopy (Genesis 150 UV-Visible Spectrophotometer, ThermoScientific), and then returned to the vial. Each UV-Vis spectrum was compared with a calibration curve to quantify the amount of epalrestat released. The calibration curve was prepared with concentrations ranging from 1 to 10 µg/mL in each of the three media; accuracy was given as 100.7% recovery and precision was given by coefficient of variation (CV) below 4%. Calibration curve in water is exemplified in Fig. S1A. Each experiment was repeated in triplicate and pictures were taken at predetermined timepoints to follow morphological changes in oleorods over time. Epalrestat solubility was determined in the three media at both 20 °C and at 37 °C by adding an excess of drug to 10 mL medium and keeping under stirring for three days. After centrifugation, epalrestat concentration in the supernatant was quantified using the corresponding calibration curve. The obtained solubility values were 8.4 (0.5) mg/L in water and SLF at 20ºC in good agreement with the chemically predicted value, and 18.1 (0.6) mg/L in water and SLF at 37ºC. In 1% Tween 80 medium the solubility increased to 80.8 (1.1) and 127.9 (1.1) mg/L at 20 and 37ºC, respectively.

### HPLC

Quantitative analysis of epalrestat concentrations lower than 10 µg/mL was performed by HPLC (Waters 717 Autosampler, Waters 600 Controller, 996 Photodiode Array Detector) with a 4.6 × 250 mm and 5 μm pores C18 Symmetry column (Waters, Ireland). The mobile phase was acetonitrile:elution buffer 45:55 (v/v). The elution buffer was composed of 25 mM potassium dihydrogen phosphate and 25 mM disodium hydrogen phosphate dihydrate in ultrapure water adjusted to pH 6.5 with phosphoric acid. The flow rate was 0.85 mL/min, the detection wavelength 295 nm, the temperature was maintained at 25˚C, and the injection volume was 40 µL. The calibration curve was prepared with concentrations ranging from 1 to 10 µg/mL with an increment step of 1 µg/mL in each of the media; accuracy was given as 100.1% recovery and precision was given by coefficient of variation (CV) below 3.4%. Calibration curve in SLF is exemplified in Fig. S1B. The retention time of epalrestat was 4.5 min with 11 min run time.

### HET-CAM

For the Hen’s Egg Test on the Chorioallantoic Membrane (HET-CAM) assay, fertilized eggs (*n* = 15) supplied by Coren (Ourense, Spain) were incubated in an CC SR 0150 incubator (Ineltec, Spain) for 9 days at 37˚C and 60% relative humidity. On the day of the experiment, the shell of the eggs was pared off with a needle and tweezers. The untouched inner membrane was moistened with a 0.9% NaCl solution and the eggs were placed back in the incubator for 30 min. The 0.9% NaCl solution was subsequently removed as well as the inner membrane while being careful not to damage the blood vessels of the CAM underneath. Any non-viable egg was discarded. NaOH 0.1 M and 0.9% NaCl were used as the positive and negative controls, respectively. Oleogels and oleorods loaded with 5% w/w epalrestat (100 µL) were placed on the CAM, and the effect on the blood vessels regarding hemorrhage, lysis and coagulation was recorded. The potential ocular irritation score was calculated with the Eq. 3 [45]:

3 $$ Irritation \; Score= \frac{301-H}{300}*5+\frac{301-L}{300}*7+\frac{301-C}{300}*9$$

### Corneal and scleral permeation

Porcine eyes were supplied by a local slaughterhouse and transported to the laboratory in diluted PBS solution at 4 ˚C in an ice bath. The eyes were obtained from healthy 6- to 8-month-old female and male pigs (Duroc, Pietrain and Belgian white pigs) weighing approximately 100 Kg. First, the corneas were dissected with 2–3 mm of surrounding sclera, and the scleras were separated from the choroid and cut into smaller pieces. Then, both tissues were washed with 0.9% NaCl and mounted in Franz diffusion cells with the outer part facing up. The area available for permeation was 0.785 cm^2^; in the case of cornea, the permeation area comprised only the central zone of the cornea without the limbus. The receiving and donor chambers were filled with 6 and 2 mL of SLF while making sure no bubbles form and then agitated with a magnetic stirring rod at 400 rpm for 1 h at 37 °C. Once the system was balanced, 0.1 mL of oleogel or oleorod (4.6 mg epalrestat) was added to the SLF solution in the donor chamber. After 30 min, at 1 h and then every hour, 1 mL of the solution in the receiving chamber was removed and replaced with 1 mL of fresh SLF. The same medium (SLF) was used in the receiving and the donor chamber as not to have transport of medium from the two chambers and measure only the transport of epalrestat. The integrity of cornea and sclera pieces was inspected visually before the start of the experiments and at the end and confirmed by the absence of any tissue damage such as cracks, cuts or opacification. The amount of drug remaining in the tissues was evaluated six hours after the start of the experiment. To do this, the tissues were weighed and incubated in ethanol at 37 ˚C for 24 h. They were then sonicated at 37 ˚C in an ultrasonic bath for 90 min. The resulting mixture was centrifuged at 1,000 rpm at 25 ˚C for 5 min and the supernatant was centrifuged at 14,000 rpm at 25 ˚C for 20 min. After filtration through 0.22 μm pore syringe filters (Scharlab, Barcelona, Spain) all the samples from the receptor chamber as well as the supernatant from the tissue incubation were analyzed by HPLC according to the protocol described above. Preliminary experiments to validate the extraction method consisted in incubating pieces of cornea and sclera in a known amount of epalrestat solution overnight, then using the extraction method to extract the epalrestat from the tissues and quantifying both the amount of epalrestat remaining in the incubation medium and the amount of epalrestat extracted from the tissue. The recovery was above 95%. No changes in the HPLC chromatogram patterns were observed between the epalrestat standard solutions and the test samples. All experiments were carried out in triplicate. The steady state flux (J) and the lag time (t_lag_) were obtained from the slope and the x-intercept, respectively, of the linear regression of cumulative amounts of epalrestat permeated per area versus time.

### IR-RAMAN study of drug penetration

Porcine cornea and sclera were mounted in Franz cells and left for 6 h in the same conditions as the permeation experiment described above. The IR-Raman study was performed by taking a minimum of 3 points and a maximum of 6 points per tissue and measuring the Raman scattering of the surface. The excitation wavelength was 532.188 nm, the sample was kept at a temperature of 8 °C during the experiment with a cooling plate, the laser power was 3 mW, and each point was measured with 60 accumulations with an integration time of 0.3 s and an objective of x50 (Zeiss LD EC Epiplan-Neofluar Dic 50x /0.55). The measurement was done on the top and bottom part of the tissue, and the absolute height of the peak (CCD cts) after smoothing and baseline correction was compared between the top and bottom of the cornea.

### Animal experiment design

The animal experiments were all performed in accordance with the Association for Research in Vision and Ophthalmology (ARVO) Statement for the Use of Animals in Ophthalmic and Vision Research [46] and with the European Directive 2010/63/EU [47]. The protocols followed 3R’s principle and were approved by the Ethics Committee for Animal Experimentation (CEEA) of the University of Santiago de Compostela (registration number: ES150780292901) and the Consellería de Medio Rural of Xunta de Galicia.

The in vivo experiments were performed on twelve healthy male New Zealand white rabbits (age approx. 3 months weighing 3.06 ± 0.20 kg). The animals were in a light-controlled room (12 h light/12 h dark cycles) at 18 °C in individual cages with unlimited access to food and water. Before animal experimentation, rabbits were carefully weighed and observed to not include rabbits with unusual low weight or corneal disruptions. No animals were discarded. During the experiments and sampling, the rabbits were maintained in restrainers with continuous monitoring to ensure there was no removal of the formulations deposited in the conjunctival sac.

To minimize the effects of subjective bias, the experiments were carried out in three days and the rabbits were randomly divided into three groups, i.e., the niosome group (*n* = 4), micelle group (*n* = 4) and oleogel group (*n* = 4). Niosomes TCD5, micelles F127 and oleogel C were used. Each rabbit received an instillation of one single drop of their respective formulation (50 µL; 0.2 mg/mL epalrestat) on the right eye. The left eyes were used as controls without treatment. Samples of lacrimal fluid were gathered before the beginning of the experiment and after instillation at predetermined timepoints along with images of the animals’ eyes. The rabbits were subsequently euthanized after 6 h and the eyes enucleated. All experiments started at 9 a.m. On the first day, the four rabbits of the niosome group were assayed. On the second day, the four rabbits of the micelle group were assayed. On the third day, the four rabbits of the oleogel group were assayed.

Prior to drug administration, the rabbits were weighed and placed into restrainers. Pictures of their eyes were taken to assess any damage or pre-existing ocular conditions. The right eye of the rabbits was used to gently instill 50 µL of the attributed formulation (0.2 mg/mL epalrestat loaded niosomes, micelles or oleogels) into the lower conjunctival sac with a micropipette while the left eye was kept as a control without treatment. Before (t = 0 h) and after the treatment instillation (t = 10 min, 20 min, 30 min and every hour until 6 h), tear fluid samples were collected from each eye by placing a Schirmer test strip in the tarsal conjunctiva of the lower eyelid for 10 s with the eye closed. The volume of the tear fluid was recorded as the millimeters of moistened strip.

### Epalrestat quantification in tear fluid

The extraction of epalrestat from the Schirmer test strips was adapted from a protocol developed by Huang et al. [48]. The strips were placed in 2 mL Eppendorf tubes with 1 mL of methanol:water 70:30 solution for 12 h at 4 °C while shaking at 50 rpm. The strips were removed, and the Eppendorf tubes were centrifuged at 14,000 rpm at 5 °C for 30 min. Finally, the supernatants were collected and stored at -80 °C until UPLC analysis. In preliminary tests that were carried out adding known volumes of known concentrations on the strips, this extraction method was shown to reproducibly recover > 98% epalrestat present in the strips. The chromatographic equipment employed was an Agilent 1290 Infinity II and QQQ G6475 mass spectrometer system. An Agilent ZORBAX Rapid Resolution High Definition (RRHD) Eclipse Plus C18 LC column (2.1 × 50 mm and particle size of 1.8 μm) was used at a flow rate of 0.5 mL/min. Water with 5 mM ammonium acetate was used as solvent A and acetonitrile with 5 mM ammonium acetate was used as solvent B. The gradient program used was as follows: 0–0.1 min. 40% B, 0.1-2.0 min. 40–100% B, 2.0-2.5 min. 100% B, 2.5–2.9 min. 100 − 60% B, and 2.9–3.0 min. 40% B. The mass spectrometer was operated in a negative ESI mode with a capillary voltage of 4400 V, a nozzle voltage of 1700 V, a gas temperature of 280 °C, a gas flow of 12 L/min and a nebulizer at 34 psi. The sheath gas temperature and gas flow were at 270 °C and at 12 L/min, respectively. The compound of interest was monitored in multiple reaction monitoring (MRM) mode. 318.0 > 273.9 m/z transition was quantified.

### Epalrestat quantification in the tissues

All rabbits were euthanized by intravenous administration of 7.5 mg/Kg of propofol and 200 mg/Kg of pentobarbital sodium (Euthasol) at the end of the experiment. Following euthanasia, the aqueous humour of both eyes of all rabbits was directly extracted from the anterior chamber using a needle and stored at -80 °C until UPLC analysis. Then, the eyes were enucleated and immediately dissected, separating and weighing the cornea, sclera, crystalline lens, vitreous humor, and retina. All tissues except the humors were treated with 1 mL of methanol for drug extraction and protein precipitation, incubated for 12 h at 4 °C and then centrifuged at 14,000 rpm at 5 °C for 30 min. After that, the supernatants were collected and MilliQ water was added to reach 30 vol% and stored at -80 °C until UPLC analysis following the protocol previously described. The addition of water was done to allow the samples to freeze at -80 °C.

### Statistical analysis

The descriptive data were presented as mean ± standard deviation. Statistical analysis, mainly one-way analysis of variance (ANOVA), was performed using Origin 2018. The level of significance was 0.05.

## Results and discussion

### Preparation of the oleogels and oleorods

Different oleogels were prepared choosing soybean oil as the main component due to its high smoke point and biocompatibility. Oleogels based on soybean oil intended for punctal occlusion have been recently shown to have an excellent ocular compatibility when applied both directly on the eye surface and when injected in the puncta [32]. Ethyl cellulose, beeswax and cocoa butter were chosen as gelators from natural, renewable sources. Beeswax and cocoa butter easily dissolved in soybean oil at 60 ºC in good agreement with the recorded DSC scans, which confirmed the melting of beeswax at 58 ºC (Fig. S2). Cocoa butter had a melting point of 19 ºC with an enthalpy of 67.53 J/g, which corresponded to polymorph I or α [49]. Differently, the semicrystalline polymer ethyl cellulose required higher heating for undergoing glass transition at 137 ºC and melting at 186 ºC (Fig. S2). Thus, oleorods preparation with ethyl cellulose required heating at 170ºC for the homogeneous dispersion of the polymer in the soybean oil. Importantly, epalrestat stability was tested by cyclic heating of the pristine drug up to 200 ºC (below its melting temperature) and then heating up to 350 ºC. The melting temperature and the melting enthalpy of preheated epalrestat (228.03 ºC, 152.6 J/g) were coincident with those of pristine epalrestat (228.07 ºC, 153.2 J/g), which confirmed thermal stability at 170 ºC (Fig. S3). It should be noted that the temperature of 170 °C was applied for oleogels/oleorods preparation only when ethyl cellulose was used. The other oleogels/oleorods were prepared by heating at 60 ºC.

**Fig. 1:**
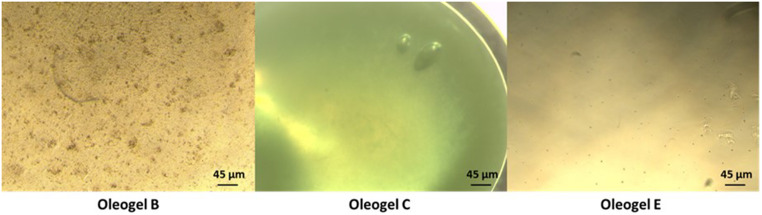
Microscope images of oleogels B, C and E (5% beeswax, 5% cocoa butter and 5% ethyl cellulose) loaded with 5% w/w epalrestat in soybean oil under x4 magnification. Scale bar 45 μm

The gelators were mixed based on Table 1 to prepare oleogels that could be extruded into oleorods, and it was found that the combination of two types of gelators for a total amount of 10% w/w gelator resulted in solid oleogels at room temperature, while a single type of gelator at 5% w/w resulted in liquid oleogels. The liquid oleogels can be administered as eye drops, while the solid oleogels can be extruded into oleorods through 22G needles. All oleogels were uniform at the macroscopic level, as depicted in Fig. 1 for oleogels made with a single gelator at 5% w/w ratio. Oleogel B made with 5% w/w beeswax was the roughest when looked at 4x magnification. Oleogel C made with 5% w/w cocoa butter was the most homogenous and kept this aspect even at 40x magnification.

**Fig. 2:**
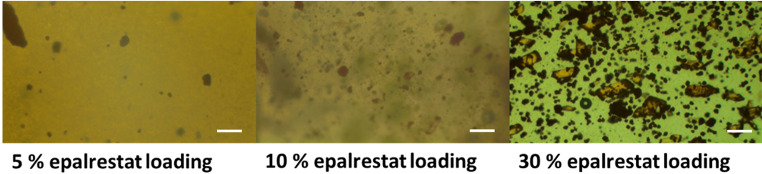
Microscope images (4x magnification) of oleorod EB, prepared mixing 5% ethyl cellulose and 5% beeswax in soybean oil and including increasing contents in epalrestat. Scale bar 45 μm

Oleorods prepared with 10% w/w gelator ratio were challenged regarding their capability to solubilize remarkably large amounts of epalrestat. Formulation EB was loaded with three different contents in epalrestat, i.e. 5%, 10% and 30% (Fig. 2), and all loading amounts resulted in an oleorod at room temperature that did not break up. Differently, when formulations EC and BC were loaded with 30% epalrestat, the resulting oleorods deposited on water broke up within one hour at 20 °C evidencing a discontinuous structure. This left pools of oil on top of the water phase. Oleorod EC was able to homogeneously incorporate 5% w/w (46 mg) and 10% w/w (92 mg) epalrestat, while oleorod BC maximum loading was 5% (46 mg) epalrestat as dissolved drug. The incorporation of different amounts of epalrestat into oleogels allowed for different levels of solubilization of the drug in the oil phase. The presence of crystals in the EB oleorods prepared with 30% epalrestat (Fig. 2) demonstrated that such high drug proportion was not able to fully dissolve within the oleogel oil phase. In subsequent experiments, 5% epalrestat loading was chosen as it would be more than enough to reach therapeutic concentrations of epalrestat [27], and it would reduce the possible irritating effects of epalrestat making the system safer. Nevertheless, higher amounts of hydrophobic drug could be loaded in oleorods if needed.

#### Viscosity

**Fig. 3:**
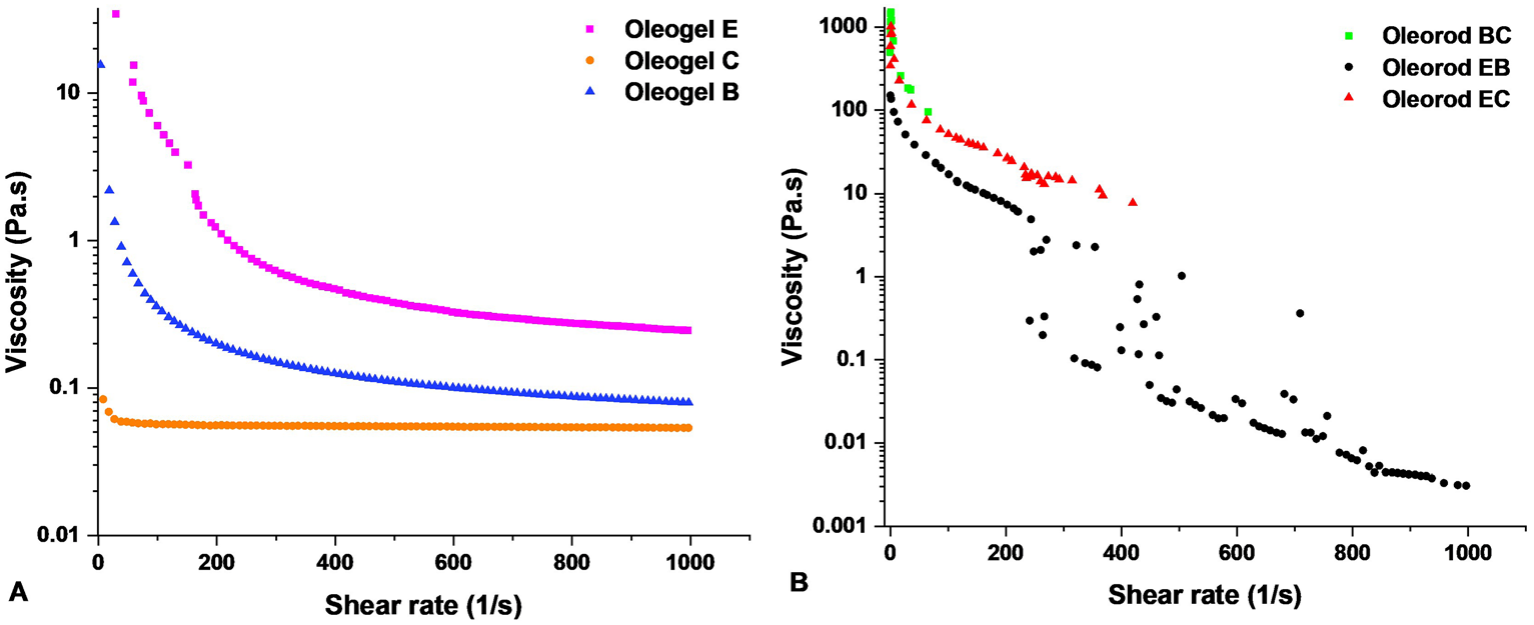
Effect of shear rate on the viscosity for oleogels comprised of 5% w/w gelator (**A**) and oleorods prepared with 10% w/w combined gelators (**B**) at 20 °C

The dynamic viscosity of the single gelator oleogels at different shear rates is displayed in Fig. 3A. There was a steep decrease of viscosity as shear rates increased for oleogels B and E while the viscosity of oleogel C remained nearly constant over the range of shear rates. Oleogel C behaved as a Newtonian fluid, with the dynamic viscosity constant over the range of shear rates, while oleogel B and oleogel E showed shear thinning behavior typical of pseudoplastic fluids [50]. When 10% w/w gelator was used, the resulting formulations showed shear thinning behavior (Fig. 3B). However, it must be noted that oleorods BC and EC did not reach the same shear rates as oleorod EB at 20 °C, and this was due to the extreme shear stresses endured by the rheometer at higher shear rates.

The viscosity values are especially important at two specific shear rates: 100 and 1000 s^-1^, which are considered to be the shear rates of the progress of the blinking movement [51]. Although the maximum shear rate of blinking can reach values as high as 20.000 s^-1^ [52], measuring the viscosity at such high shear rates is redundant as the Newtonian plateau is reached at lower values [53]. To keep precorneal residence, the viscosity of the ocular formulation needs to be above 0.01 Pa.s [54, 55], which all oleogels achieved in the range of blinking shear rates as shown in Table 2. However, it is important to notice that formulations E, EB, EC and BC had viscosities at 100 s^-1^ that could be uncomfortable in the context of topical administration. Within the double gelator formulations, only oleorod EB reached low viscosity values at high shear rates due to the strong pseudoplastic behavior displayed. The viscosity of oleogel C (Table 2) corresponded closely to the viscosity of soybean oil at the upper shear rate of the blinking process. Commercial artificial tear solutions commonly have viscosities between 1 and 10 Pa.s when behaving as Newtonian fluids, and between 8 and 100 Pa.s in the 100 s^-1^ to 1000 s^-1^ shear rate range when exhibiting shear thinning behavior [53]. A positive correlation has been established between higher viscosity and longer precorneal retention both in rabbit and humans [54, 56]. The optimal eye drop is therefore viscous enough to increase its precorneal retention time while simultaneously still being able to be administered in drop form. On their part, oleorods made of binary combinations of gelators may be suitable for ocular insert development.

**Table 2:** Viscosity (Pa·s) of the formulations at 100 and 1000 s^− 1^ at 20 °C

Shear rate (s^− 1^)	E	B	C	EB	EC	BC	Soybean oil
100	6.013	0.359	0.0566	17.10	51.2	-	0.612
1000	0.245	0.079	0.0535	0.0031	-	-	0.056

The most suitable oleogel for eye drops (oleogel C) and the aqueous-based formulations chosen as reference for the in vivo tests were also investigated regarding their viscosity at 35ºC (Fig. S4). Niosomes TCD5 prepared as previously reported [11] had mean size of 79.88 nm, PDI 0.51, zeta-potential of + 15.67 ± 8.53 mV, and a loading efficiency of 99.76 ± 0.35 (%). Micelles F127 had mean size of 19.30 nm, PDI 0.69, zeta-potential of + 0.79 ± 4.46 mV, and a loading efficiency of 98.55 ± 0.64 (%); values that agreed well with previous publications [33]. When comparing oleogel C to the aqueous-based nanocarriers formulations (Fig. S4), the oleogel was more viscous. Oleogel C and the micelles reached a viscosity plateau (at 0.035 and 0.015 Pa.s respectively) around 60 and 200 s^-1^ respectively. In contrast, the niosomes displayed more pronounced shear thinning behavior, but lower viscosity overall, starting at 0.019 Pa.s and reaching a plateau of 0.0013 Pa.s at a shear rate of 450 s^-1^. Niosomes behave as pseudoplastic as the interactions among large vesicles oppose to initial flow. This behavior is in accordance with the current literature [11, 57, 58]. The micelles were kept at low temperature (4 ºC) during the whole preparation process and storage to ensure that no gel was formed. The sol-gel transition temperature for micelles made from 10 mM Pluronic^®^ F127 is 35 °C [33], which explains the higher viscosity of the micelles when compared to the niosomes. The oleogel C behaved in a nearly Newtonian way, with a small viscosity decrease past the very low shear rate interval, in good agreement with previous reports on other oleogels [59].

#### Epalrestat release

Oleorods were made by extruding 0.1 mL of oleogel through a 22G needle and directly deposited on top of an aqueous subphase. The release of epalrestat from the oleorods was carried out over different time periods ranging from a couple of hours to several days, in DI water, SLF and 1% v/v Tween 80 aqueous solution. The release on SLF was performed to mimic the clinical practice where the topically applied formulation would have to release epalrestat to the tear fluid. Differently, the release on a 1% v/v Tween 80 aqueous solution phase was done to simulate conditions with high solubility of epalrestat in aqueous solution and avoid reaching a false plateau due to medium saturation. A volume of release medium of 10 mL was chosen to facilitate drug dissolution in the medium (poorly soluble) and to mimic the rapid dilution of the topically applied formulations on the eye surface. The release was also tested at 20 °C and at 37 °C to mimic storage and administration conditions. The volume of the oleorods was 0.1 mL in all cases, meaning the total amount of epalrestat available for release per oleorod was 4.6 mg.

**Fig. 4:**
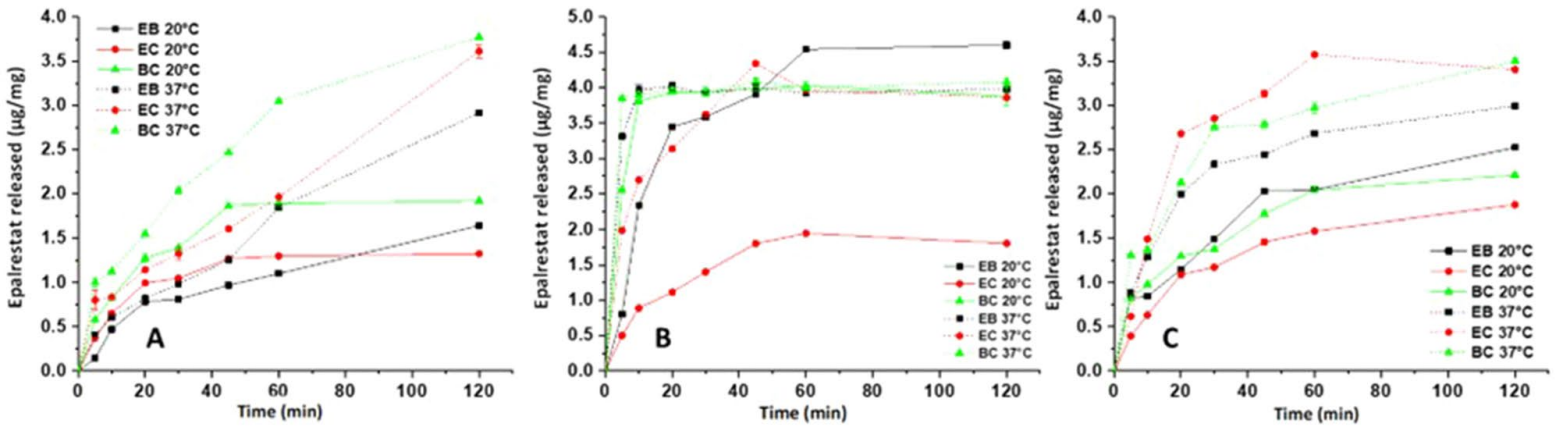
Drug released from oleorods loaded with 5% w/w epalrestat at 20 and 37 °C deposited on top (**A**) DI water for 2 h, (**B**) SLF for 2 h, (**C**) 1% Tween 80 v/v aqueous solution for 2 h (*n* = 3)

First the release of epalrestat from the oleorods was tested at 20 °C in DI water, SLF and 1% Tween 80 v/v aqueous solution for 2 h. As shown in Fig. 4A and Fig. S5, epalrestat got released at 20 °C into distilled water at a higher rate from the start of the experiment to 45 min from oleorods EC and BC, then plateaued at 1.3 µg/mg (3.0% of total epalrestat loading) and 1.9 µg/mg (4.4% of total epalrestat loading), respectively. This was also the behavior of oleorod EB (3.8% of total epalrestat loading released at 2 h), with the exception of the plateau where it instead had a linear release profile. Differently, the release of epalrestat at 37 °C was linear for all formulations. The increase of epalrestat released at 37 °C (EB: 6.7%; EC: 8.3%; BC: 8.7% of total epalrestat loading) is likely due to the increased solubility of epalrestat at higher temperatures.

The release of epalrestat in SLF followed a burst release pattern with the exception of oleorod EC at 20 °C (Fig. 4B). The plateau of epalrestat released was the same (3.90 µg/mg; 9% of total epalrestat loading) for oleorod EB at 37 °C and oleorod BC at both temperatures and reached after 10 min. Differently, oleorod EB at 20 °C released rapidly until 20 min and then slower but at constant rate until plateauing from one hour on. Epalrestat got released from oleorod EC at 37 °C rapidly in 10 min and then linearly until 45 min, reaching the same plateau as oleorods EB and BC at 37 °C. For topical administration, the burst release pattern shown by oleorods EB and BC is favorable as ocular clearance will remove the formulation from the ocular surface even when deposited into the conjunctival sac.

The release into 1% Tween 80 v/v aqueous solution was faster at 37 than 20 °C (Fig. 4C and S5). The release of epalrestat into 1% Tween 80 v/v aqueous solution was rapid until 30 min, where the release rate diminished. The amounts of epalrestat released after 2 h where approx. 3.4 µg/mg and 3.5 µg/mg (7.8% and 8.0% of total epalrestat loading respectively) corresponding to oleorods EC and BC at 37 °C. This is surprising as the expectation was that the total release of epalrestat in 1% v/v Tween 80 aqueous solution would be significantly higher than in DI water or SLF. This slow-release behavior correlates well with the high consistency (viscosity) of the oleorods, which may regulate drug diffusion through a slow erosion process which is not dependent on the solubility of the drug in the release medium.

**Fig. 5:**
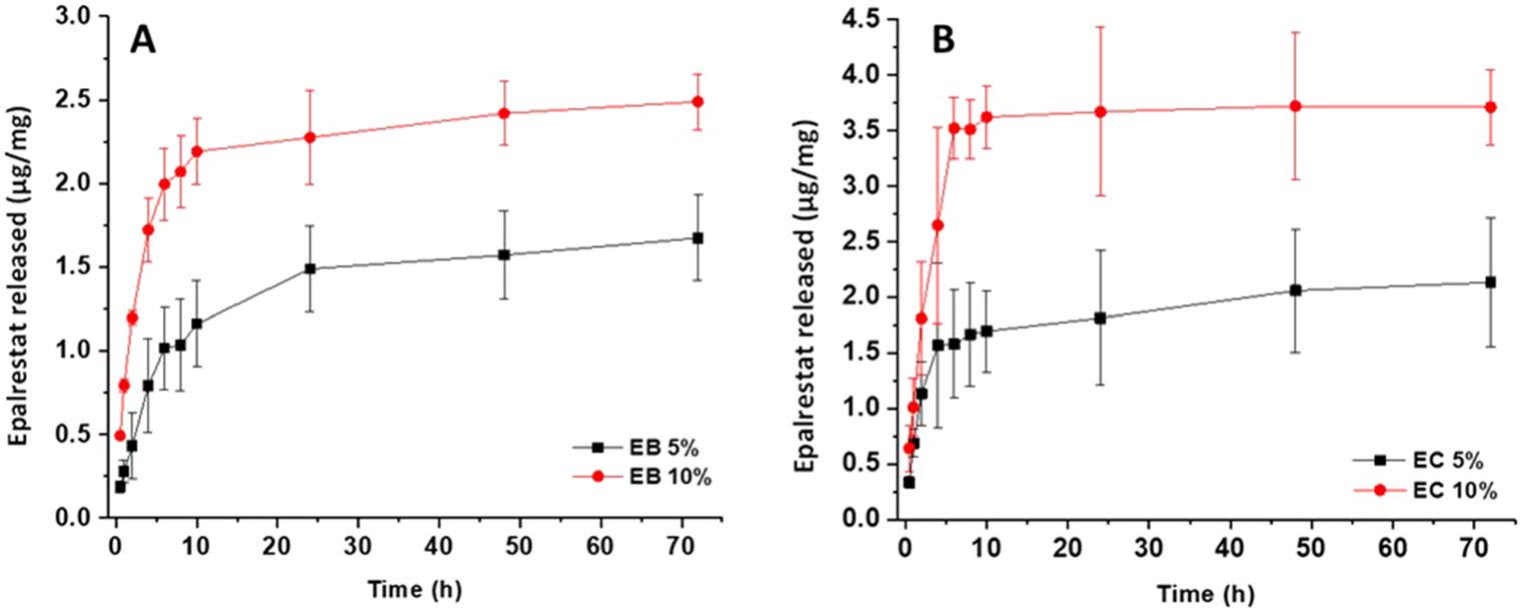
Epalrestat release from oleorod EB (**A**) and oleorod EC (**B**) at 20 °C in SLF over 3 days

The release of epalrestat in SLF from EB (ethyl cellulose/beeswax) and EC (ethyl cellulose/cocoa butter) oleorods was also measured when the oleorods were loaded with different amounts of drug (Fig. 5). The release from oleorods loaded with 5 and 10% epalrestat plateaued after 24 h for EB oleorods and 10 h for EC oleorods disregarding the content in epalrestat (Fig. 5). However, as expected, the oleorod loaded with 10% epalrestat released about twice the amount of the oleorod loaded with a 5%.

The in vitro experiments allowed comparing the capability of different formulations for controlling drug release under certain well-controlled conditions, but prediction of the in vivo behavior from the in vitro release data is very difficult as there are many intrinsic variables (blinking rate, blinking pressure on the formulation, tear flow,…) that still cannot be mimicked in vitro [38]. While measuring the release profile of epalrestat from oleorods in 10 mL is important to understand the limitations of drug release, such a release volume is not realistic, as the tear film volume passing on the ocular surface is at most 1 mL in 6 h [60, 61]. Furthermore, during topical administration of oleogels, the deposition of the formulation is likely to be in drop format, restricting the extrusion of oleogels to volumes closer to 50 µL. The release of epalrestat was higher at 37 °C than at 20 °C in each release medium, as expected, due to the higher solubility of epalrestat at higher temperatures [62, 63]; namely, in 1% Tween 80 medium the solubility increased to 80.8 (1.1) and 127.9 (1.1) mg/L at 20 and 37ºC (measured by us). The most remarkable result was the change in release behavior when the oleorods were deposited on top of a SLF solution, where a small burst of epalrestat occurred. This release behavior of the hydrophobic epalrestat into an ionic medium is in accordance with the results of Buyukozturk et al. [64] where naproxen would also release from soybean oil emulsions into PBS at 37 °C at a fast rate. The content of epalrestat in the oleorods was of 4.6 mg so the amounts released over 2 h were always under 10%, but it is well known that drug release from oleogels typically takes time, with the release of diclofenac from paraffin oleogels reaching 7.34% after one hour [65], or the release of ciprofloxacin reaching 10% from Span 60/mustard oil oleogels [66]. Oleorods prepared by Macoon et al. intended for intraocular injection displayed release times of 125 days to release 85% of the dexamethasone loaded in the device [26].

As shown in Fig. S6, the release of epalrestat was made evident by the color gradient of the oleorod, shifting to a less saturated color with time. Moreover, the oleorod showed a change in the morphology of the rod structure over 4 days and sectioned into multiple smaller rods over time, increasing surface area further. According to Macoon et al. [28], oleogel inserts composed of 10% w/w β-sitosterol/lecithin (8:2) and 15% w/w sorbitan monostearate gradually dissolve, achieving complete dissolution after 2 and 5 months, respectively. This contrasts with oleorods made from 5% w/w beeswax, which maintained their structure even after 110 days. The smaller overall size of the oleogel inserts (2–4 µL) compared to those extruded in our study (0.1 mL) may explain the absence of sectioning, as each section in image E of Fig. S6 was approximately the size of the oleorod extruded in Macoon et al.‘s study. Due to the high cost of epalrestat and the release limit of the drug in these conditions, formulations loaded with 5% w/w epalrestat loading were chosen for the following experiments.

#### HET-CAM

The HET-CAM assay was performed to predict the ocular irritation that the oleogels may cause. This assay is not considered an animal experiment under Directive 2010/63/EU [47] and gives an accurate idea of the safety of the formulations due to the similarity found between the CAM and the human conjunctiva [45]. The binary oleorods (EB, EC and BC) tested lost their shape after being brought in contact with the CAM at 37 ºC (Fig. S7). Signs of hemorrhage, lysis or coagulation were not noticed, rendering an ocular irritation score of 0, similar to the negative control. This suggests that the oil-based formulations developed are safe for topical administration to the eye. Moreover, the formulations protected the vasculature of the CAM membrane from the damaging effects of epalrestat, since when the drug was tested in its free form it led to an ocular irritation score of 18.58 at 0.2 mg/mL concentration [11]. When compared to the results of HET-CAM assays of epalrestat-loaded niosomes previously developed by our group, oleogels were able to protect the CAM to the same extent as niosomes [11]. Differently, the positive (0.1 M NaOH) control displayed an ocular irritation score of 19.5 ± 0.26.

#### Ex vivo corneal and scleral permeation

**Fig. 6:**
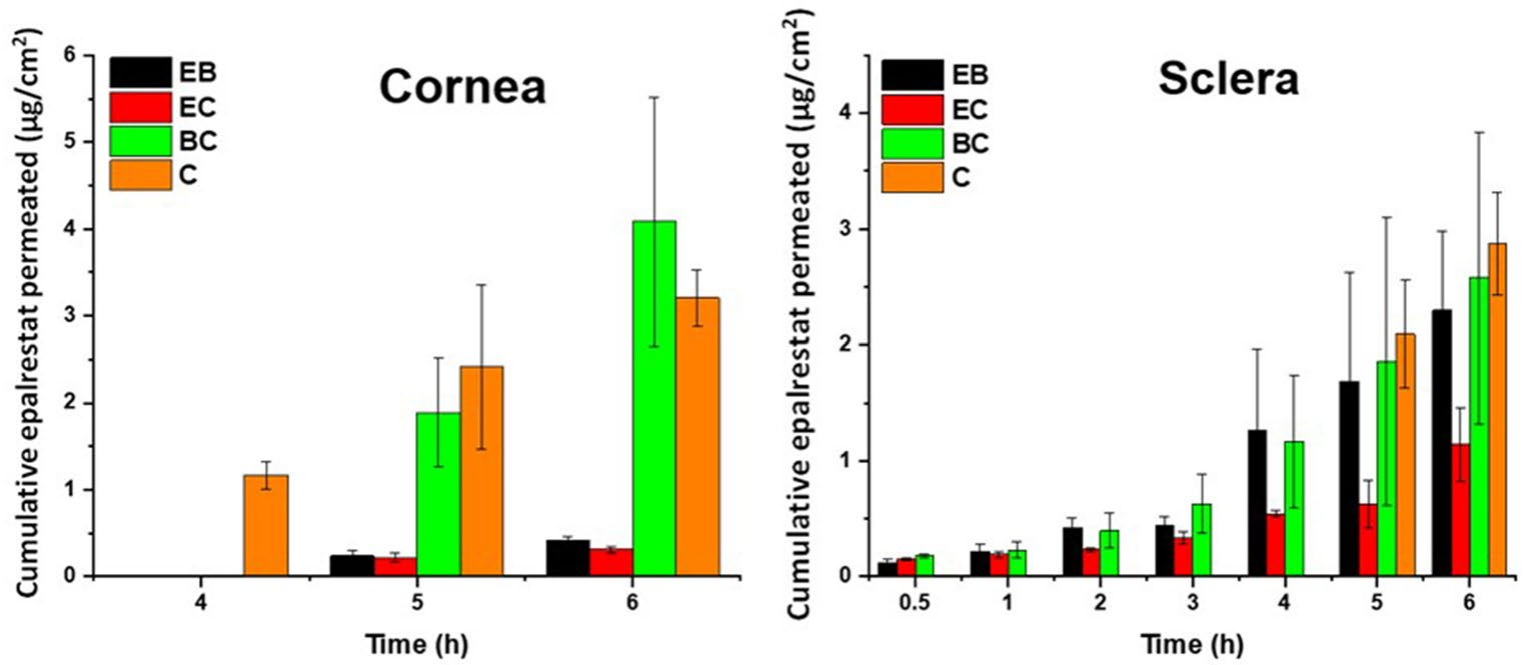
Amounts of epalrestat permeated through cornea and sclera during 6 h permeation experiments in Franz’s diffusion cells when delivered from oleorods EB, EC and BC, and oleogel C (all loaded with 5% epalrestat)

During the experiments the oleorods floated on the surface of the medium of the donor chamber. Thus, there was no direct contact of the formulation with the ocular tissues. The corneal permeation of epalrestat from the oleorods only started after 4 h of contact as can be seen in Fig. 6. Oleorod BC outperformed oleorods EB and EC by permeating about 8 times more by 6 h. Differently, for scleral permeation, epalrestat started to permeate from 30 min on, with all four formulations permeating linearly. Oleorods BC and EB outperformed oleorod EC at 6 h, but by a smaller margin than for corneal permeation. Oleogel C favored epalrestat permeation in sclera and cornea providing mean values similar to those of oleorod BC but with more homogeneous values (smaller standard deviations) which could be related to its lower viscosity and thus more accurate administration. Epalrestat concentrations in the donor chambers after 6 h experiment were 34.5 (s.d. 15.9), 96.5 (s.d. 18.5), 225.7 (s.d, 67.9), 38.7 (s.d.29.4) µg/mL for oleorods EB, EC, BC and oleogel C, respectively. The corneal and scleral permeation of epalrestat from oleogel C loaded with 0.2 mg/mL epalrestat (the concentration used for the animal experiments) can be seen in Fig. S8.

**Fig. 7:**
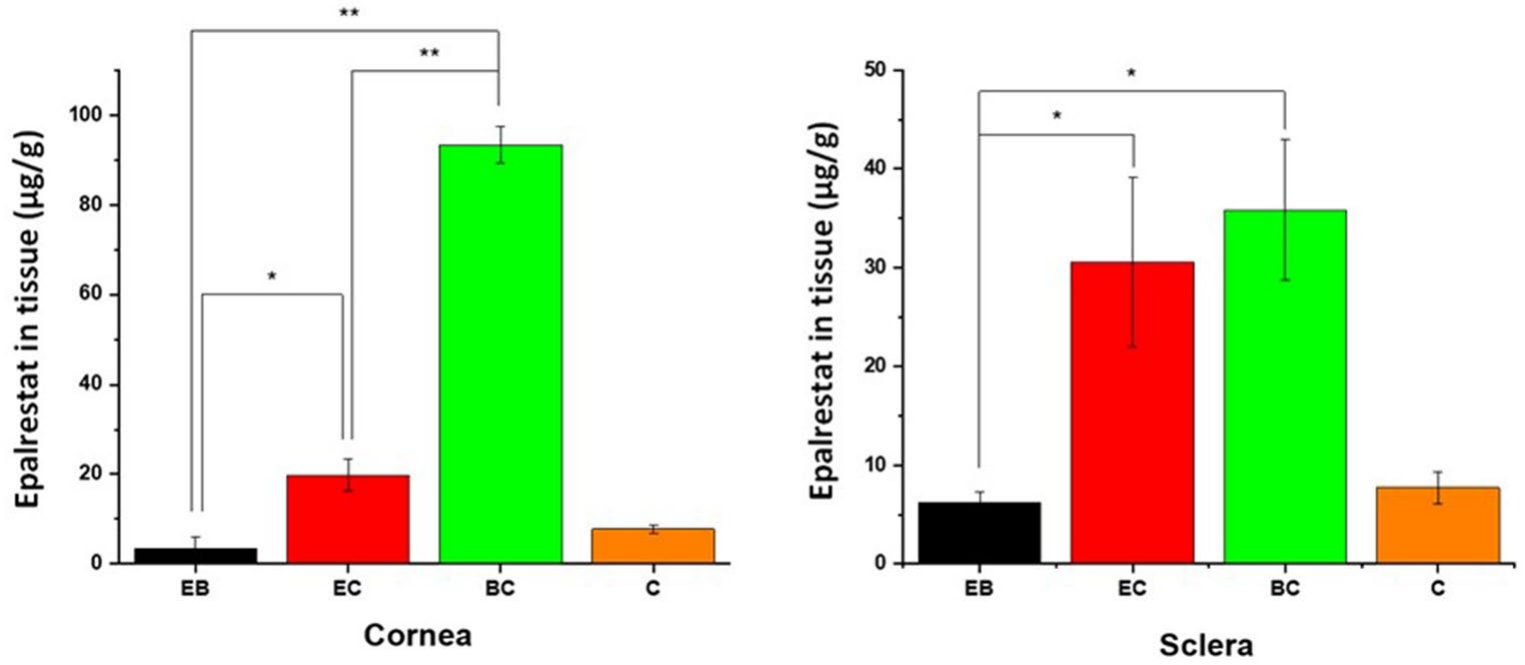
Epalrestat retained in corneal (left) and scleral (right) tissues after 6 h permeation experiment in Franz’s diffusion cells. *Statistically significant higher concentrations in the tissue (*p* < 0.05), **Statistically significant higher concentration in the tissue (*p* < 0.01)

The amount of epalrestat accumulated in the tissues after 6 h of permeation was quantified by HPLC (Fig. 7). In both corneal and scleral permeation studies, oleorod BC (93 µg/g and 36 µg/g) accumulated more epalrestat in the tissue than oleogels EC (20 µg/g and 31 µg/g) and EB (3 µg/g and 6 µg/g), which is in line with the permeation data and the higher epalrestat concentration in the donor chamber. For oleogel C the amounts of epalrestat retained in both tissues were similar (7.76 µg/g in cornea and 7.72 µg/g in sclera). The permeability coefficients of epalrestat delivered through each tissue by each oleorod are summarized in Table 3. The permeability coefficient of epalrestat across porcine cornea and sclera was calculated as the ratio of the steady state flux and the concentration of the donor chamber at six hours. The linear regressions for oleorods EB, EC and BC for the cornea were done with only two data points and therefore the results should be taken with caution.

**Table 3:** Steady state flux, lag time and permeability coefficient of oleorods EB, EC and BC, and oleogel C for corneal and scleral tissues

Sample		Steady State Flux (µg/cm^2^ × h)	Lag Time (min)	Permeability Coefficient (×10^6^ cm/s)
Cornea	EB	1.307 ± 0.912	402.4 ± 246.3	2.520 ± 0.481
	EC	0.573 ± 0.093	161.0 ± 40.2	0.796 ± 0.130
	BC	13.19 ± 5.931	246.1 ± 15.3	18.31 ± 8.238
	C	1.021 ± 0.299	134.0 ± 20.2	11.58 ± 6.019
Sclera	EB	2.196 ± 1.547	61.90 ± 25.8	3.050 ± 2.100
	EC	0.949 ± 0.254	20.70 ± 4.99	1.318 ± 0.350
	BC	2.571 ± 1.809	35.60 ± 17.5	3.571 ± 2.500
	C	0.778 ± 0.245	138.6 ± 26.2	4.596 ± 1.398

Overall, the permeation of epalrestat through corneal and scleral tissues was successful. The permeability coefficients of the corneal permeation were very different for each oleorod, which could be explained by the long time needed for the drug to start being detected in the receptor chamber of the Franz cell. The permeability coefficients were therefore calculated with linear regressions relying on less data points than the coefficients obtained for scleral permeation. As a trend, the non-aqueous formulations prepared using cocoa butter solely (oleogel C) or combined with beeswax (oleorod BC) were those that most favored the access of epalrestat to the cornea and to a lesser extent to the sclera. Remarkably, the interaction with the cornea was notably promoted after few hours in contact, which is different to what drug-loaded nanocarrier aqueous dispersion usually favors (commonly the transscleral route). This finding is in good agreement with the more favorable interaction between the oily excipients and the lipophilic cornea epithelium compared to the more hydrophilic sclera tissue [17].

The permeability coefficients of epalrestat from previously developed niosomal formulations were 0.63 ± 0.40·10^6^ cm/s in cornea and 0.75 ± 0.15·10^6^ cm/s in sclera [11]. Thus, epalrestat formulation in oleorod/oleogel containing beeswax provided higher permeability, while the amounts accumulated in the tissues were similar to those observed for oleogel C. However, it needs to be pointed out that the 5% w/w loading of the oleorods represent 4.6 mg total epalrestat in the donor chamber, while in the study with niosomes the amount of epalrestat in the donor was 0.4 mg. This means 11.5 times increase of available epalrestat in the oleogel formulation. When relativized in regard to the amount of epalrestat in the donor, the oleorods BC (the most efficient ones) were more effective in promoting cornea penetration (2.52-fold) and less effective in promoting sclera penetration (0.41-fold). This would mean that the permeation of epalrestat through corneal and scleral tissues follow Fickian diffusion kinetics and is affected by saturation effects in the donor chamber, and therefore, limited solubility of epalrestat in the donor medium may limit the drug available for permeation. This hypothesis is further supported by the results of ex vivo corneal permeation of ciprofloxacin from graphene oxide reinforced nanocomposite oleogels that managed to reach cumulative permeation percentages between 0.6% and 1.0%, with cumulative drug releases between 0.7% and 1.2% [29]. The Fickian diffusion of the drug was also the determining factor for the corneal permeation of voriconazole from palmitic acid and safflower oil oleogels, even though the drug molecule was more hydrophilic than epalrestat or ciprofloxacin and was able to reach cumulative drug permeations values as high as 35% [31].

### IR-RAMAN imaging

**Fig. 8:**
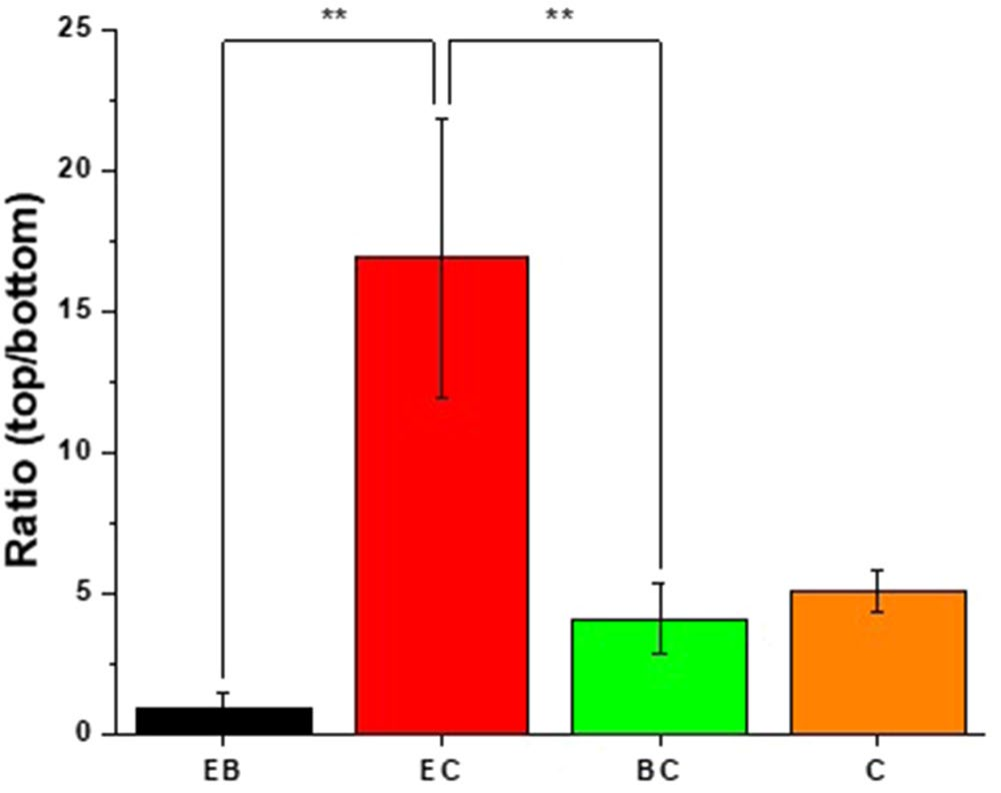
Ratio of the top to bottom intensity of Raman peaks indicating the presence of epalrestat in the cornea’s outside layers. **Statistically significant higher top to bottom ratio (*p* < 0.01)

IR-RAMAN spectroscopy was used to assess the presence of epalrestat at the top and bottom parts of the cornea after 6 h of contact to confirm the permeation results displayed in the previous section (Fig. 8). Oleorod EB showed a ratio of top to bottom close to 1, confirming permeation of epalrestat through the cornea, with equal epalrestat presence on both sides of the tissue. The top to bottom ratio of oleorod EC was the highest, meaning that there was more epalrestat present on the donor chamber side of the tissue compared to that of the receiver chamber side of the tissue, in good agreement with the low permeability of epalrestat when delivered using this oleorod. The top to bottom ratio of oleogel C being 5.1 indicates a behavior similar to oleorod BC.

The top to bottom ratios of the different oleogels indicate differences in the amounts of epalrestat in the first few microns of the cornea in contact with each of the chambers of the Franz cell. Interestingly, both in the recovery of epalrestat from permeated tissue and IR-RAMAN data, oleorod EC displayed higher values than oleorod EB. The ratio close to 1 of oleogel EB is logical seen as the epalrestat recovered from the tissue (3.45 µg/g) was significantly smaller than the epalrestat recovered from oleogels EC and BC (19.79 µg/g and 93.37 µg/g respectively). The high corneal permeation of epalrestat in the case of oleogel BC is corroborated by the high amount of epalrestat recovered in the corneal tissue and further supported by the ratio of 4 between the epalrestat concentration on top and on the bottom of the cornea, pointing to an epalrestat gradient in the tissue. However, the permeation behavior of oleogel EC is less evident, as it was the oleogel with the highest top to bottom ratio (17), but with a recovery of 19.37 µg/g epalrestat in the cornea and a cumulative amount of epalrestat permeated of 0.31 µg/cm^2^ after 6 h. A possible explanation could be that oleorod EC at 37 °C started to dissolve [67] and the hydrophobic polymer ethylcellulose was deposited on the corneal tissue, creating a barrier to permeation which would explain the poor permeability results and the high ratio of epalrestat concentration from top to bottom. The ratio of oleogel C would indicate a higher concentration of epalrestat in the top layers of the cornea than in the bottom layers, signifying that the permeation of epalrestat through the tissue had not reached equilibrium after 6 h.

### In vivo tests

#### In vivo experiment

The in ovo assay previously carried out indicated that all formulations developed here were capable of efficiently protecting tissues from the irritating effects of epalrestat so, an in vivo experiment was planned with a threefold objective: (I) confirm the safety of the formulations deposited on the ocular surface of rabbits, (II) quantify the amounts of epalrestat in the lacrimal fluid over the duration of the experiment, and (III) quantify the accumulation of epalrestat in the different ocular tissues after 6 h. This information is essential to ensure safety and efficiency of the formulations in the next steps of development, trending towards clinical trials. During the experiment the right eye was always the eye receiving the formulation while the left eye was kept as a control. The images displayed in Table S1 show that there was no ocular damage induced by the formulations. The formulation that irritated the rabbits the most was oleogel C where the conjunctiva gets progressively more red over the course of the experiment. According to the European Community guidelines for the use of in vivo rabbit eye test [68] and the European Union Dangerous Substances Directive (EU DSD) [69] the presence of minimal or reversible irritancy does not eliminate a formulation from eligibility for clinical evaluation, but quantitative data would need to be gathered to assess the severity of the irritancy in the case of the oleogel. The lacrimal concentration data from two rabbits (Rb2 and Rb3) in the niosomes group as well as one rabbit (Rb7) from the micelle group were not used as the values were not reasonably believable and probably came from contamination of the samples. The data from the aqueous humor from the right eye of one niosome group rabbit (Rb1) and from the aqueous humor of the left eye of one oleogel group rabbit (Rb 11) were not used as the values were not reasonably believable and probably came from contamination of the samples.

#### Epalrestat quantification in the lacrimal fluid

**Fig. 9:**
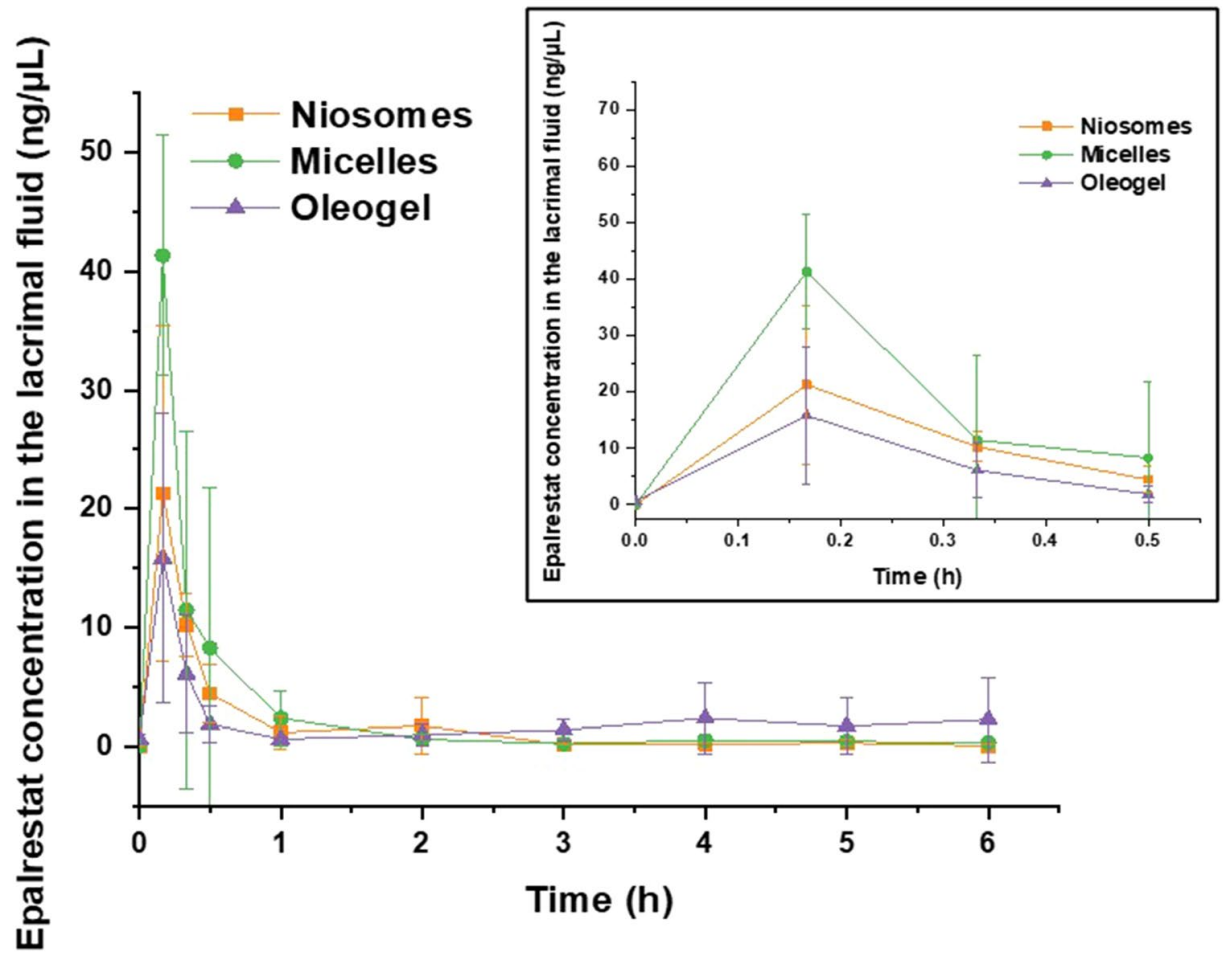
Epalrestat concentration in tear fluid after a single instillation of epalrestat-loaded niosomes TCD5, micelles F127 and oleogel C (50 µL, 0.2 mg/mL, *n* = 4 for each formulation) (mean values and standard deviations)

Each of the three formulations had their epalrestat concentration peak measured 10 min after instillation and then the concentration gradually decreased up to ~ 2 h (Fig. 9). The micelle formulation reached a higher concentration in lacrimal fluid (48.3 ± 16.2 ng/µL) than the niosomes (12.3 ± 13.2 ng/µL) and the oleogel (15.8 ± 12.2 ng/µL). But interestingly, the values for the lacrimal concentration of epalrestat in the case of the oleogel from 3 to 6 h were higher than the ones from micelles and niosomes, which trended towards zero, indicating that the oleogel deposited in the conjunctival sac did not completely get cleared by the tear turnover. No significant differences were found in the epalrestat lacrimal concentration during the 6 h of the experiment. The seemingly higher lacrimal concentration of epalrestat when encapsulated in F127 micelles could be explained by the formation of a gel at 35 °C. The temperature of the eye [70] is approximatively the sol-gel transition temperature of the micellar dispersion [33], which would increase its retention time compared to niosomes. For the oleogel, its higher viscosity might have led to longer retention of the gel in the conjunctival sac, creating a depot that would release the drug in the later stages (3–6 h) of the experiment.

#### Epalrestat in the tissue

**Fig. 10:**
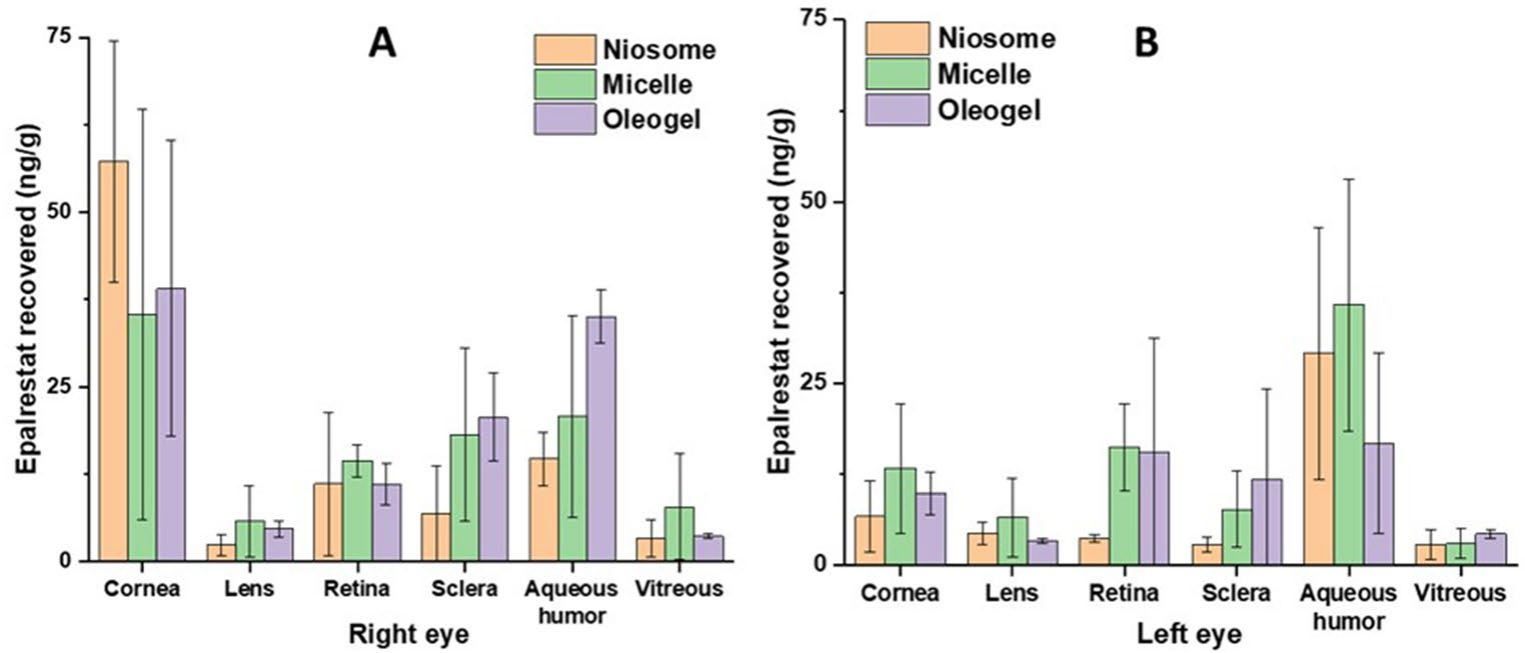
Epalrestat accumulated in various ocular tissues of (**A**) right and (**B**) left eyes 6 h after eye drop (50 µL) instillation (*n* = 4)

Biodistribution of epalrestat over the ocular tissues of both eyes was evaluated 6 h after instilling one single eye drop (50 µL) of the corresponding formulation on the right eye of the rabbits (Fig. 10). The highest amounts of epalrestat were recovered from the cornea and the aqueous humor of the oleogel groups, respectively. Since the cornea is in direct contact with the formulation, the high concentration of epalrestat in this tissue indicates that the drug was able to permeate inside the tissue from the tear film. Moreover, the high amount of epalrestat also found in the aqueous humor indicates that the drug was capable of passing through the cornea, however it was then blocked from diffusing further. The low amounts of epalrestat found in the vitreous and in the lens suggest that the scleral route was favored for drug delivery to the posterior segment of the eye. The scleral route was targeted when the topical formulations were designed, and the comparison of epalrestat found in the retina and in the sclera point to scleral permeation without significant retention. Indeed, the drug transport pathways are governed by pressure differentials going from the back to the front of the eye [71]. The values of epalrestat concentration in the left eyes were lower than those of the eyes where the formulations were administered, with the unexpected exception of the aqueous humor recorded for micelles and niosomes. While the high amounts of epalrestat recovered from the retina of the control eyes can be explained by the high vascularization of the tissue [72] which could receive epalrestat from the bloodstream, the high presence of epalrestat in the aqueous humor of the control eye is less evident.

To the best of our knowledge this is the first study that compares the ocular biodistribution of epalrestat from three different formulations: niosomes, micelles and oleogels. Permeation assays in vivo with oleogels are still incipient. Cao et al. have evaluated cyclosporin A [32] distribution in the cornea, conjunctiva and sclera when an organogel made from stearic acid, soybean oil, and N-methyl-2-pyrrolidinone was deposited in the lacrimal canaliculi to block lacrimal drainage. The concentrations of cyclosporin A at 6 h were higher when the organogel was used compared to an ophthalmic solution, and the concentration ranges for the cornea were around 150 ng/g for the cornea and 40 ng/g for the sclera. In the case of a drug suspension of voriconazole, the drug concentration in the aqueous humor decreased significantly after only 2 min, and even with the use of a proniosomal in situ gelling ocular insert, the concentration of drug decreased significantly after 8 min [73]. Another proniosomal gel study investigating the distribution of lomefloxacin reported a significantly higher lomefloxacin concentration in aqueous humor, vitreous humor, cornea and conjunctiva when compared to a marketed formula. This was due to the mucoadhesive properties of the proniosomal gel, and the non-ionic surfactants acting as penetration enhancers [74]. The penetration enhancement from non-ionic surfactants would also explain the higher corneal concentration of epalrestat in the right eye of the rabbits found here.

## Conclusion

Oleogels are being investigated as drug delivery platforms, the information on their potential as platforms for ocular drug administration is still limited. To the best of our knowledge, this is the first study incorporating epalrestat into oleogels for ocular delivery. Epalrestat being a highly hydrophobic molecule, delivering it to the different tissues of the eye is complicated. This study shows the successful preparation of oleogels and oleorods encapsulating epalrestat resulting in a system that allows for sustained release of epalrestat. Oleogels facilitated corneal and scleral permeation, without causing relevant ocular irritation and promoting in vivo ability to reach inner eye tissues. No significant differences were found among the oleogels and the two nanocarrier-based aqueous formulations (niosomes and micelles); all of them being able to deliver epalrestat to the different tissues of the eye in vivo. However, since oleogels and oleorods can be loaded with remarkably higher concentrations of epalrestat, the use of these non-aqueous formulations may be advantageous when the disease requires higher drug concentrations. The safety of formulations loaded with high contents in epalrestat require further assessment.

## Electronic supplementary material

Below is the link to the electronic supplementary material.

Supplementary Material 1. Calibration curves of epalrestat; DSC scans of the excipients and epalrestat; DSC scans of epalrestat before and after heating at 200 ºC; Effect of shear rate on the viscosity for niosomes TCD5, micelles F127 and oleogel C at 35 °C; Drug released from oleorods loaded with 5% w/w epalrestat at 20 °C and 37 °C; Oleorod EB loaded with 10% w/w epalrestat releasing the drug on DI water at 20 °C; Pictures of the chorioallantoic membrane after 5 min contact; Amounts of epalrestat from the oleogel C loaded with 0.2 mg/mL epalrestat permeated through cornea and sclera; and Images of the eyes of the rabbits taken before (t = 0 h) and after (t = 1, 4 and 6 h) administration of niosomes TCD5, micelles F127 and oleogel C

## Data Availability

The datasets generated and/or analyzed during the current study are available from the corresponding author on reasonable request.
