# Supplementary material for: Oleogels for the ocular delivery of epalrestat: formulation, in vitro, in ovo, ex vivo and in vivo evaluation
Source: Drug Deliv Transl Res. 2024 May 23;14(11):3291–308. doi: 10.1007/s13346-024-01560-7 (PMC11445291; doi:10.1007/s13346-024-01560-7)
Supplement: Supplementary file 1 — Supplementary Material 1. Calibration curves of epalrestat; DSC scans of the excipients and epalrestat; DSC scans of epalrestat before and after heating at 200 ºC; Effect of shear rate on the viscosity for niosomes TCD5, micelles F127 and oleogel C at 35 °C; Drug released from oleorods loaded with 5% w/w epalrestat at 20 °C and 37 °C; Oleorod EB loaded with 10% w/w epalrestat releasing the drug on DI water at 20 °C; Pictures of the chorioallantoic membrane after 5 min contact; Amounts of epalrestat from the oleogel C loaded with 0.2 mg/mL epalrestat permeated through cornea and sclera; and Images of the eyes of the rabbits taken before (t = 0 h) and after (t = 1, 4 and 6 h) administration of niosomes TCD5, micelles F127 and oleogel C [file 13346_2024_1560_MOESM1_ESM.pdf]

Supporting information

Oleogels for the ocular delivery of epalrestat: formulation, *in vitro*, *in ovo*, *ex vivo* and *in vivo* evaluation

Axel Kattar[1], Maria Vivero-Lopez[1], Angel Concheiro[1], Rajeev Mudakavi[2], Anuj Chauhan[2], Carmen Alvarez-Lorenzo [1*]

[1] *Departamento de Farmacología, Farmacia y Tecnología Farmacéutica, I+D Farma Group (GI-1645), Facultad de Farmacia, Instituto de Materiales (iMATUS) and Health Research Institute of Santiago de Compostela (IDIS), Universidade de Santiago de Compostela, 15782 Santiago de Compostela, Spain*

[2] *Department of Chemical Engineering, Colorado School of Mines, Golden, CO 80401, United States*

*Corresponding author: Carmen Alvarez-Lorenzo; email : carmen.alvarez.lorenzo@usc.es

Supporting information

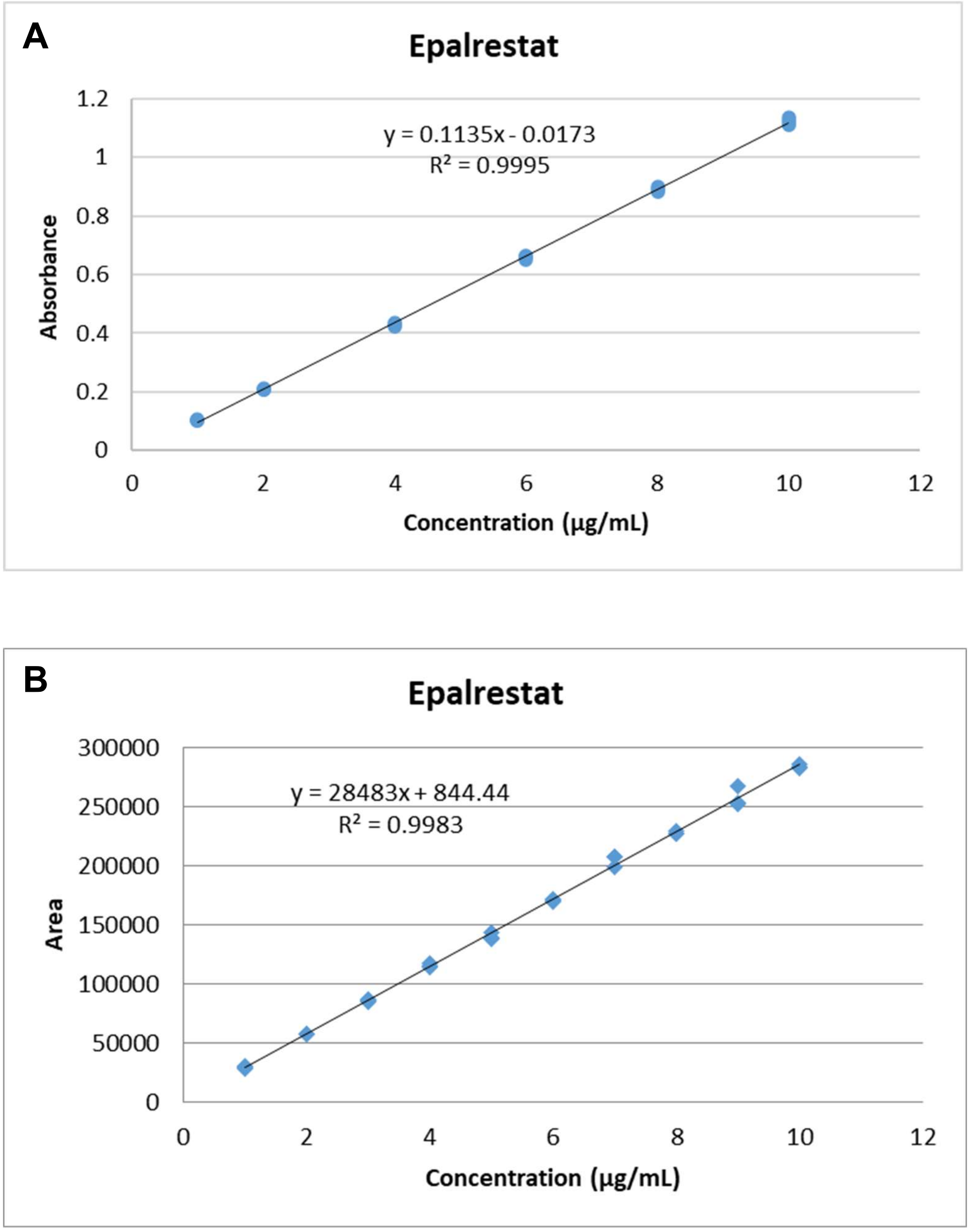

Figure S1. Calibrations curves of epalrestat (A) in water as determined at 400 nm by UV-vis spectrophotometry and (B) in SLF as determined at 295 nm by HPLC.

Supporting information

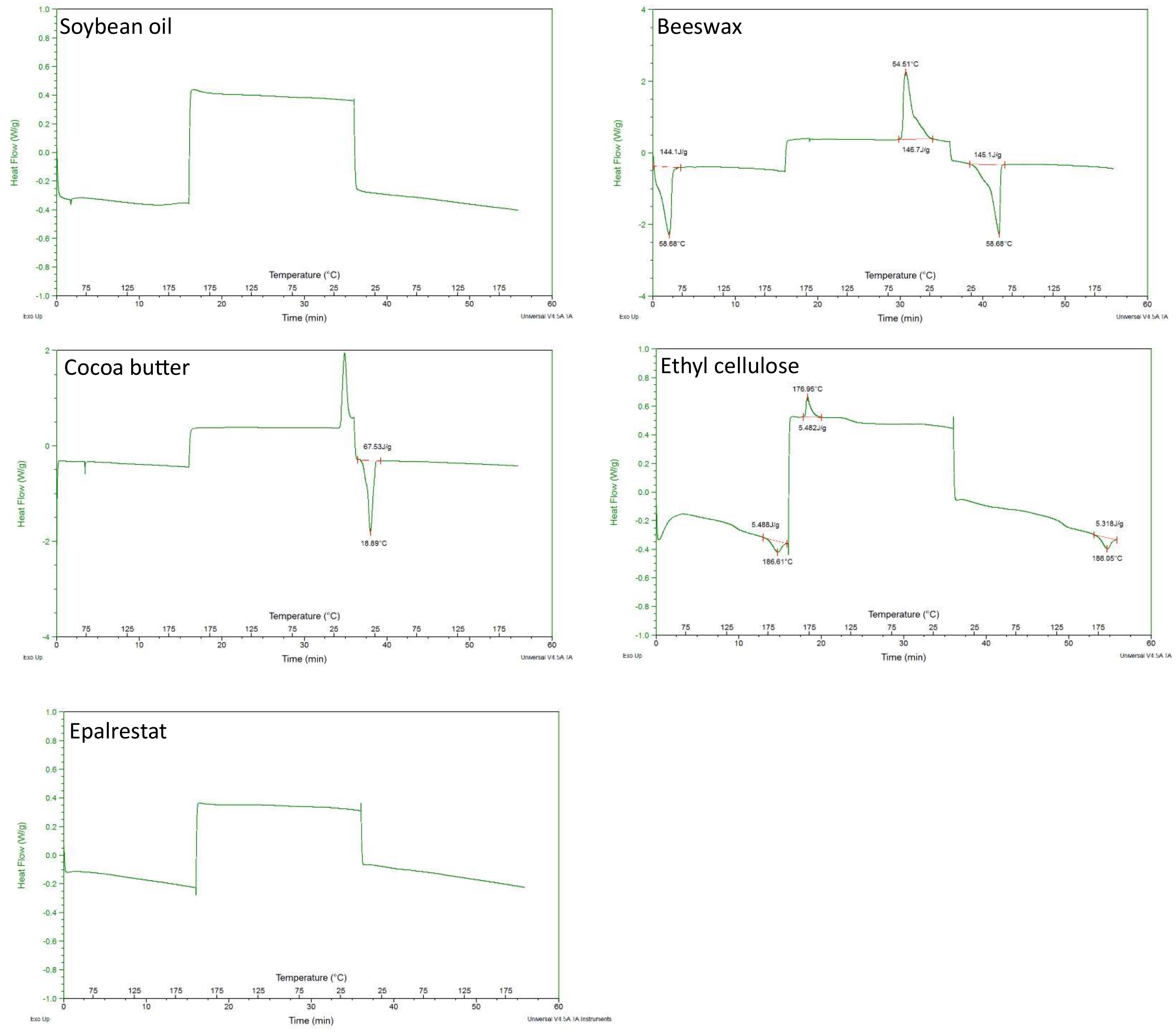

Figure S2. DSC scans of the excipients and epalrestat recorded by heating samples (1-3 mg) in aluminum pans (with lid) from 40ºC to 200ºC, cooling to 0 ºC, and heating again up to 200ºC, at 10 ºC/min.

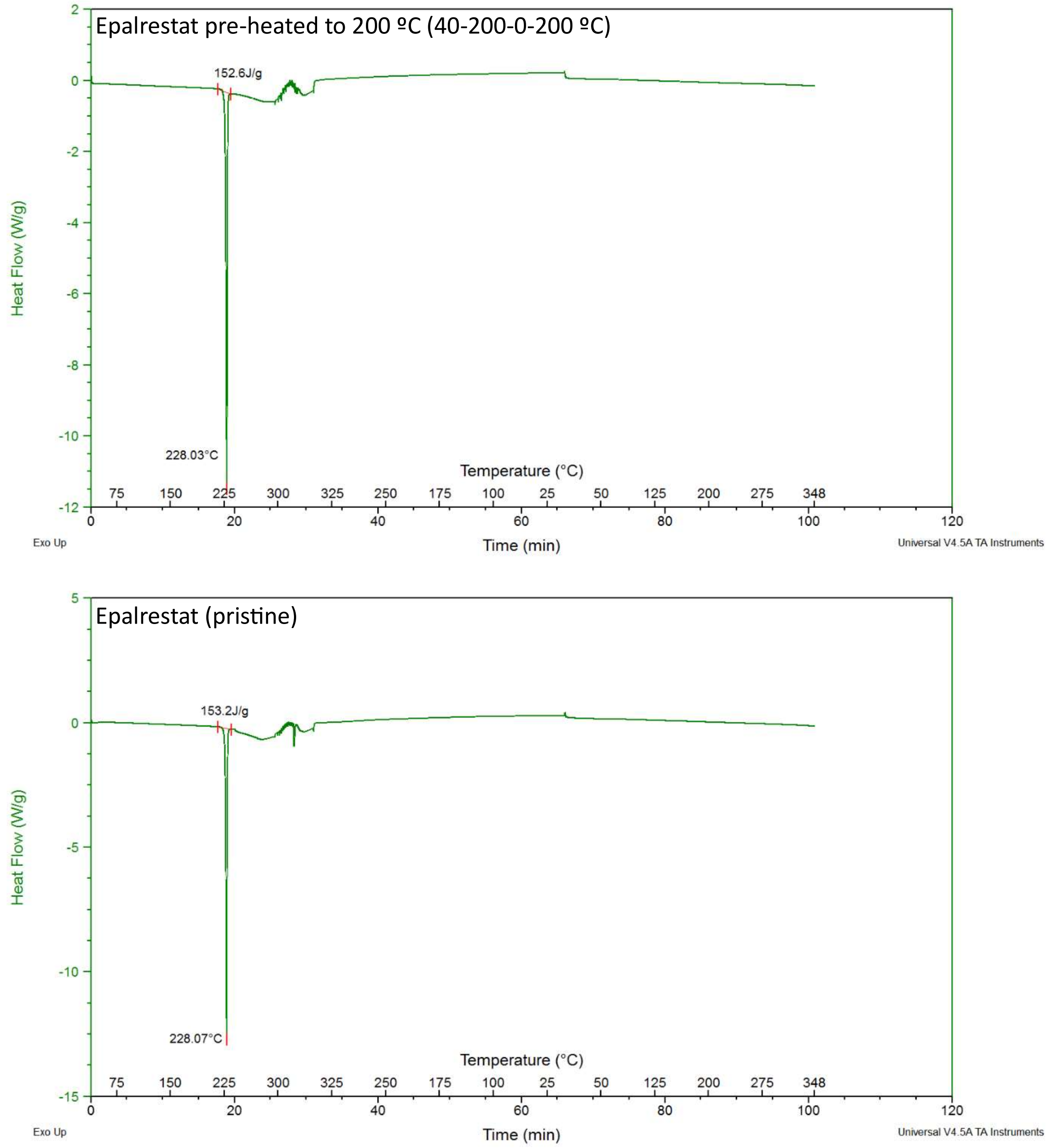

Figure S3. DSC scans of epalrestat recorded by heating samples (1-3 mg) in aluminum pans (with lid) from 40ºC to 350ºC, cooling to 0 ºC, and heating again up to 350ºC, at 10 ºC/min. The melting temperature and the melting enthalpy were similar for pristine epalrestat and for epalrestat that was cyclically preheated up to 200 ºC (40-200-0-200 ºC).

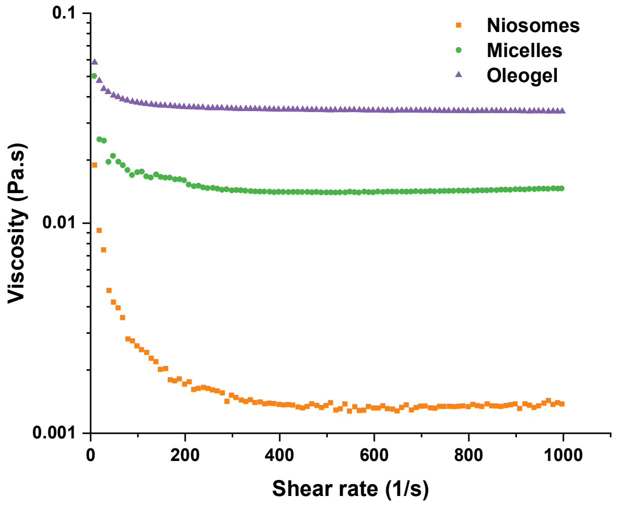

Figure S4. Effect of shear rate on the viscosity for niosomes TCD5, micelles F127 and oleogel C at 35 °C.

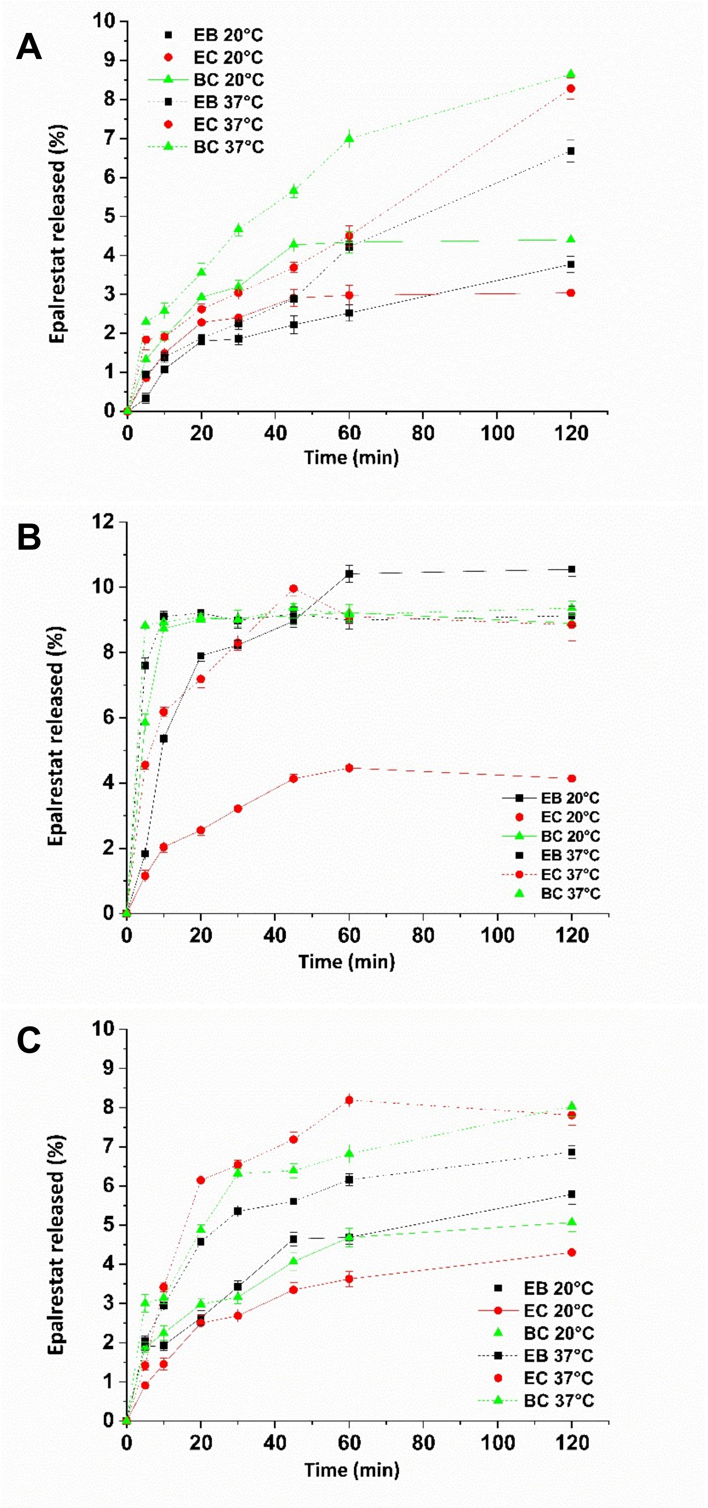

Figure S5. Drug released from oleorods loaded with 5% w/w epalrestat at 20 °C and 37 °C deposited on top (A) DI water for 2 h, (B) SLF for 2 h, (C) 1% Tween 80 v/v aqueous solution for 2 h (n=3).

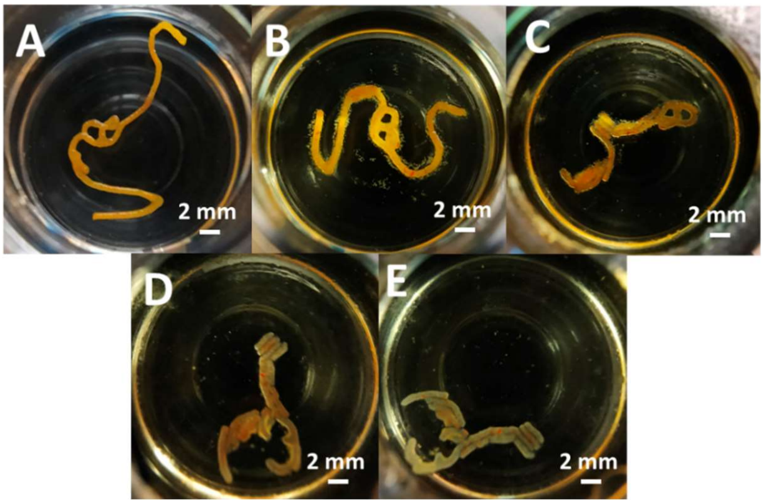

Figure S6. Oleorod EB loaded with 10% w/w epalrestat releasing the drug on DI water at 20 °C. The images were taken at timepoints: A =0; B = 1h; C = 6h; D = 1 day; E = 4 days at equivalent distance from the surface of the water.

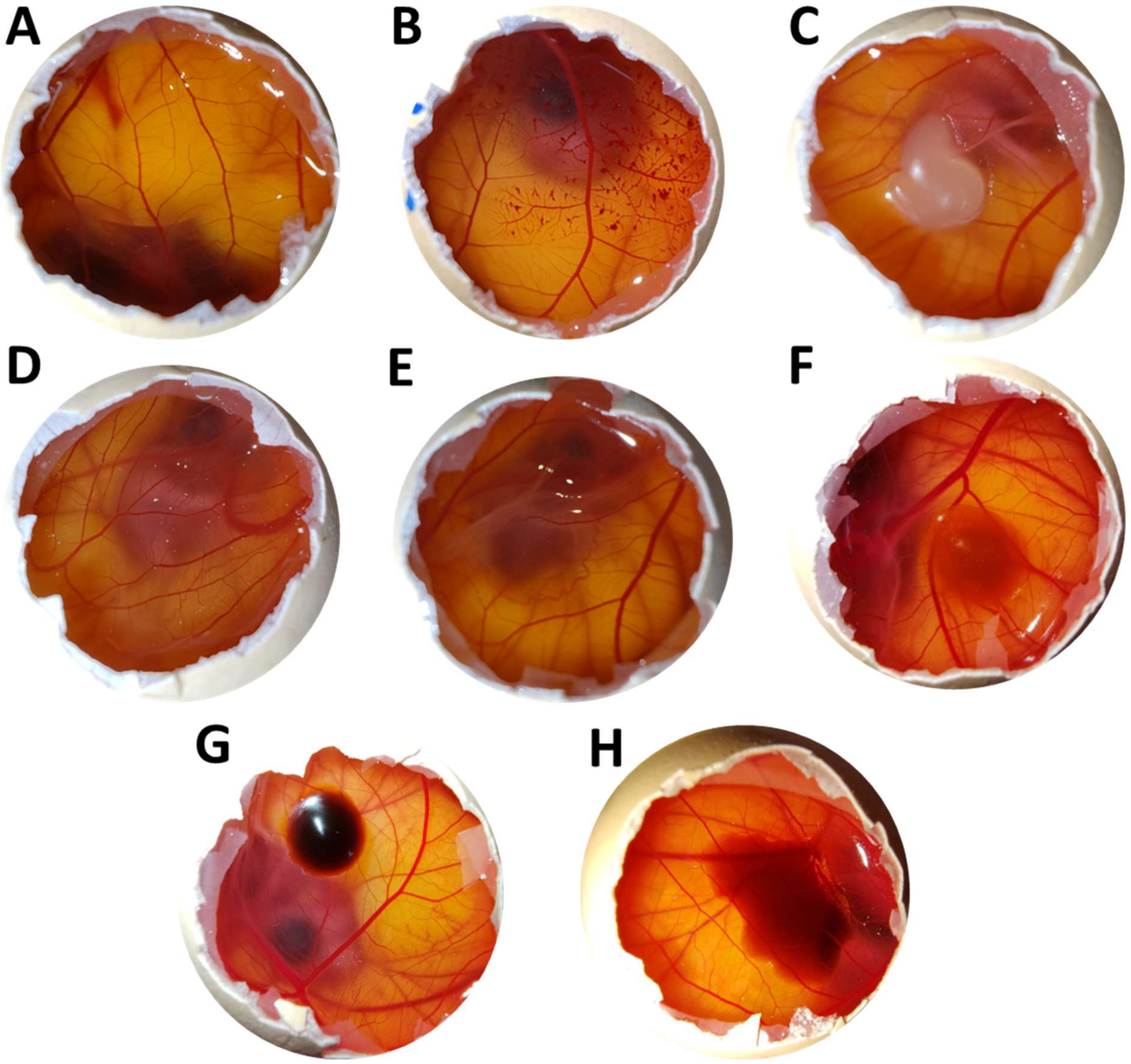

Figure S7. Pictures of the chorioallantoic membrane after 5 min contact with A: Negative control (0.9% NaCl), B: Positive control (0.1 M NaOH), C: oleogel B, D: oleogel E, E: oleogel C, F: oleorod EB, G: olerod EC, and H: oleorod BC. All formulations were loaded with 5% w/w epalrestat.

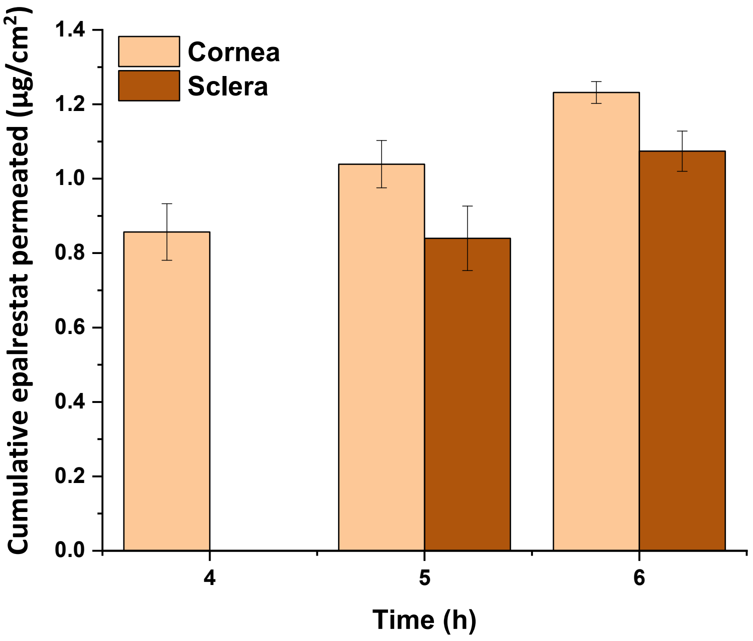

Figure S8. Amounts of epalrestat from the oleogel C loaded with 0.2 mg/mL epalrestat permeated through cornea and sclera during 6 h permeation experiments in Franz's diffusion cells.

Table S1. Images of the eyes of the rabbits taken before (t = 0 h) and after (t = 1, 4 and 6 h) administration of niosomes TCD5, micelles F127 and oleogel C loaded with 0.2 mg/mL epalrestat to the right eye.

**Niosomes**	**Right eye**	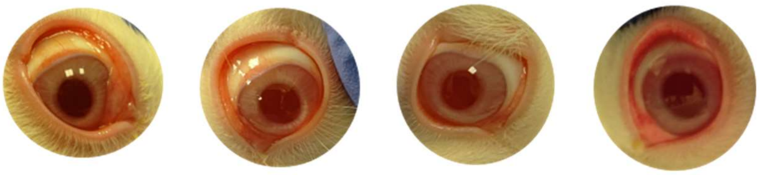
	**Left eye**	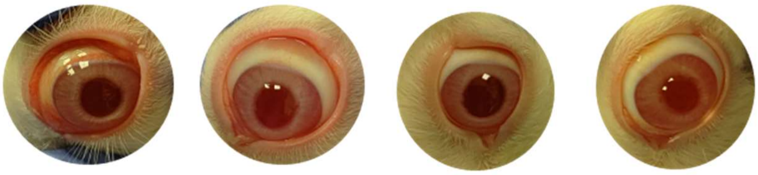
**Micelles**	**Right eye**	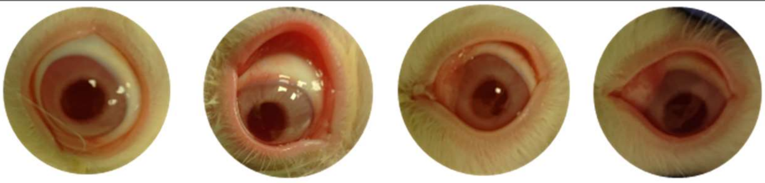
	**Left eye**	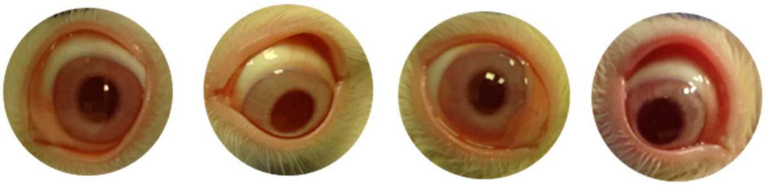
**Oleogel**	**Right eye**	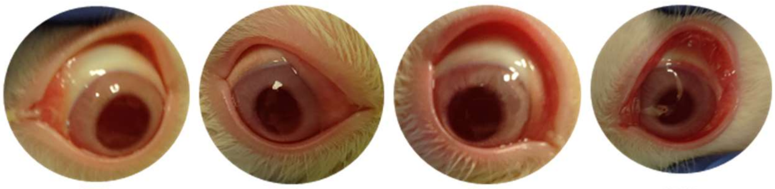
	**Left eye**	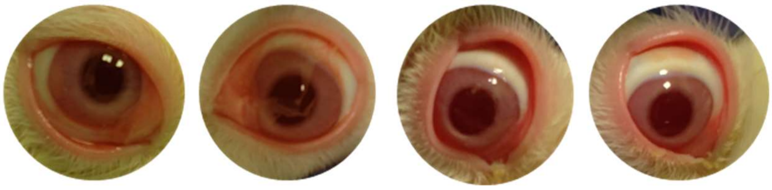
